# *Heyndrickxia coagulans* IDCC 1201 Alters Gut Microbiome and Metabolome in Patients with Functional Bowel Disorders

**DOI:** 10.3390/nu18152466

**Published:** 2026-07-29

**Authors:** Hyeon Ji Jeon, Jin Seok Moon, Hye Min Jeong, Won Yeong Bang, Hayoung Kim, Donggyu Kim, Minhye Shin, Jungwoo Yang, Jongbeom Shin, Young Hoon Jung

**Affiliations:** 1 School of Food Science and Biotechnology, Kyungpook National University, Daegu 41566, Republic of Korea; hhyyeeoonnji@knu.ac.kr (H.J.J.); jeonghyemin0534125@gmail.com (H.M.J.); 2 ILDONG Bioscience Co., Ltd., 17 Poseunggongdan-ro, Poseung-eup, Pyeongtaek-si 17957, Republic of Korea; moonjs@ildong.com (J.S.M.); yeong0417@ildong.com (W.Y.B.); young@ildong.com (H.K.); 3 Department of Microbiology, College of Medicine, Inha University, Incheon 22212, Republic of Korea; ehdrb1548@inha.edu (D.K.); mhshin@inha.ac.kr (M.S.); 4 Department of Microbiology, College of Medicine, Dongguk University, 123 Dongdae-ro, Gyeongju 38066, Republic of Korea; dbl3jwy@dongguk.ac.kr; 5 Division of Gastroenterology, Department of Internal Medicine, Inha University Hospital, Inha University School of Medicine, Incheon 22212, Republic of Korea

**Keywords:** *Heyndrickxia coagulans*, functional bowel disorders, gut microbiome, fecal metabolomics, probiotics

## Abstract

**Background/Objectives:** Functional bowel disorders (FBDs) are chronic gastrointestinal conditions that substantially impair quality of life. This randomized, double-blind, placebo-controlled trial investigated the effects of *Heyndrickxia coagulans* IDCC 1201 (COA 1201) in adults with FBD. **Methods:** Participants received COA 1201 or a placebo for 8 weeks, with outcomes assessed using the irritable bowel syndrome (IBS) Symptom Severity Score (IBS-SSS), IBS Quality of Life (IBS-QOL), and bowel activity measures. And Fecal microbiome and metabolomics were measured by 16S rRNA gene sequencing gas chromatography-mass spectrometry, respectively. **Results:** Both groups showed improvement from baseline, but COA 1201 produced greater symptom relief, particularly in abdominal bloating, post-defecation discomfort, and the body image domain of IBS-QOL. Fecal microbiome profiling revealed modest changes in global diversity, yet taxa linked to saccharolytic activity and short-chain fatty acid production—including *Ruminococcus bromii*, *Agathobacter rectalis*, and *Bifidobacterium*—were enriched in participants receiving COA 1201, whereas *Clostridium leptum* increased in those receiving a placebo. Untargeted metabolomics demonstrated distinct metabolic signatures between groups, confirmed by supervised partial least squares discriminant analysis. Exploratory metabolites were identified using variable importance in projection scores (>1.5), statistical significance (*p* < 0.05), and absolute log_2_ fold change (>1). Alanine and proline emerged as time-dependent metabolites, while tryptophan, lactic acid, and phytosphingosine were specifically associated with COA 1201 treatment. **Conclusions:** Collectively, these findings suggest that COA 1201 alleviates FBD symptoms by modulating the gut microbiome and metabolome.

## 1. Introduction

Functional bowel disorders (FBDs) encompass a spectrum of chronic gastrointestinal conditions characterized by abdominal pain, bloating, distension, and irregular bowel habits in the absence of structural abnormalities [1]. According to the Rome IV criteria, FBDs include irritable bowel syndrome (IBS), functional constipation (FC), functional diarrhea (FD), functional abdominal bloating (FAB), and functional abdominal distension (FAD) [2]. These disorders impose a substantial burden on quality of life and healthcare systems worldwide, affecting up to 40% of adults, depending on the diagnostic approach and region [3,4]. Despite their high prevalence, FBDs remain challenging to manage due to their multifactorial pathophysiology and heterogeneous clinical presentation.

Emerging evidence indicates that FBDs arise from complex interactions involving altered gut motility, visceral hypersensitivity, intestinal barrier dysfunction, immune dysregulation, and gut microbiota imbalance [1,5]. In particular, low-grade inflammation and subtle immune activation, rather than overt mucosal damage, are increasingly recognized as key contributors to symptom generation [6]. Dysbiosis has been consistently reported in FBDs, with microbial alterations linked to changes in metabolic outputs that influence motility, epithelial integrity, and gut–brain signaling [7,8]. Reduced or atypical microbial diversity has also been associated with constipation and diarrhea [9,10,11]. Consequently, microbiota-targeted interventions have attracted considerable interest as therapeutic strategies, although responses vary across individuals and microbial subtypes [12].

Probiotics—defined as live microorganisms that confer health benefits when administered in adequate amounts—have demonstrated modest but clinically meaningful effects in FBDs, particularly in alleviating abdominal pain, bloating, and bowel habit irregularities [13,14]. However, outcomes remain inconsistent across studies, largely due to strain-specific differences in biological activity, survivability in the gastrointestinal tract, and mechanistic engagement with host physiology [15]. This underscores the need for well-characterized probiotic strains supported by robust preclinical evidence before clinical evaluation. 

*Heyndrickxia coagulans* (formerly known as *Bacillus coagulans*) is a spore-forming probiotic with practical advantages over non-spore-forming lactic acid bacteria, including enhanced stability, resistance to gastric acidity, and improved viability during storage and gastrointestinal transit [16]. Among its strains, *H. coagulans* IDCC 1201 has been isolated from green malt and proven safe through extensive genomic, phenotypic, and toxicological characterization [17]. Enteric delivery systems have further enhanced its stability and enabled targeted intestinal release, reinforcing its translational potential for clinical applications [18,19]. 

Beyond safety and formulation, *H. coagulans* IDCC 1201 has demonstrated biologically relevant functions aligned with key pathophysiological features of FBDs. Preclinical studies have shown that this strain suppresses inflammatory responses in macrophages and exhibits antibacterial activity against pathogenic microorganisms, suggesting potential to modulate immune signaling and microbial interactions within the gut environment [20]. These properties are particularly relevant to FBDs, where immune activation and dysbiosis are believed to contribute to persistent symptoms despite the absence of overt inflammation [21]. In addition, while several *Bacillus* species are utilized as probiotics due to their spore-forming ability, *H. coagulans* uniquely combines spore formation with lactic acid production, contributing to both environmental tolerance and gut health [22].

Preclinical studies further support the functional relevance of *H. coagulans* IDCC 1201 to bowel disorders. In a constipation model, formulations containing *H. coagulans* IDCC 1201 improved bowel movement frequency and stool parameters, indicating a beneficial effect on intestinal motility [23]. Additionally, in a colitis model, this strain ameliorated inflammatory responses and protected intestinal integrity [24]. Although these models cannot fully replicate the complexity of FBDs, they provide mechanistic and functional evidence supporting clinical translation of this strain for disorders involving altered motility, barrier function, and low-grade inflammation. Nevertheless, clinical data remain limited, particularly regarding integrated assessments of symptom improvement alongside gut ecosystem function such as fecal metabolomics. Microbial metabolites—including short-chain fatty acids (SCFAs), amino acid derivatives, and neuromodulatory compounds—play pivotal roles in mediating host–microbiota interactions that influence intestinal motility, barrier function, and mucosal immunity, thereby contributing to symptom onset and exacerbation [25,26]. Accordingly, clinical trials evaluating *H. coagulans* IDCC 1201-based formulations are essential to determine efficacy while simultaneously characterizing microbiome and metabolomic changes, thereby establishing evidence-based protocols for FBD management.

In this study, we conducted a randomized, double-blind, placebo-controlled clinical trial in adults with FBDs to assess the effects of *H. coagulans* IDCC 1201 (COA 1201) on gastrointestinal symptoms and quality of life. In parallel, fecal microbiome profiling and untargeted metabolomic analyses were conducted to explore potential microbial and metabolic mechanisms underlying symptomatic improvement.

## 2. Materials and Methods

### 2.1. Ethics and Informed Consent

This study was conducted at Inha University Hospital (Incheon, Republic of Korea) in accordance with the Declaration of Helsinki and International Conference on Harmonization Good Practice (ICH-GCP) guidelines. The protocol was approved by the Institutional Review Board of Inha University Hospital (IRB No. 2023-10-016), and the follow-up study was additionally approved by the same Institutional Review Board (IRB No. 2026-05-012). Also, It was registered in the Clinical Research Information Service (CRIS) database [Registration ID: KCT0011004; first registration date: 10 September 2025]. Written informed consent was obtained from all individuals prior to any study-specific procedures.

### 2.2. Study Design and Participants

A randomized, double-blind, placebo-controlled, parallel-group clinical trial was conducted in adults aged 19–70 years who met the Rome IV diagnostic criteria for at least one FBD, including FC, FD, FAB, or FAD. A total of 39 participants were initially assessed for eligibility, of whom 1 was excluded due to withdrawal of consent (*n* = 1). The remaining 38 participants were randomized into the Test group (*n* = 19) and Placebo group (*n* = 19). During the trial, 0 participants dropped out in the Test group, whereas 4 participants dropped out in the Placebo group due to principal investigator termination instructions (*n* = 1), concomitant use of prohibited medication (*n* = 2), and withdrawal of consent (*n* = 1). Consequently, a total of 34 participants (Test group, *n* = 19; Placebo group, *n* = 15) completed the study per-protocol and were included in the downstream 16S rRNA sequencing-based metagenomics and GC-MS-based metabolomics analyses (Figure S1). 

Exclusion criteria included: acute gastrointestinal infections; peptic ulcer disease; significant uncontrolled chronic illness; history of gastrointestinal (GI) surgery other than appendectomy and cesarean section; recent (within 2 weeks) use of medications or supplements affecting GI function; pregnancy or breastfeeding; known allergies to investigational product components; severe psychiatric disorders (e.g., major depressive disorder, schizophrenia); history (within 2 years) of ulcerative colitis, Crohn’s disease, celiac disease, colorectal cancer; and prior enrollment in another clinical trial within 3 months.

Sample size was calculated using G*Power (version 3.1.9.7; Heinrich-Heine-Universität Düsseldorf, Düsseldorf, Germany), assuming an effect size of 0.80 and 80% power to detect between-group differences in the primary outcome at week 8. Allowing for a 20% dropout rate, the final target sample size was 19 participants per group. 

Eligible participants were randomized in a 1:1 ratio to receive *H. coagulans* IDCC 1201 (COA 1201) or placebo using a computer-generated sequence. Allocation was concealed using sequentially numbered, identical containers prepared by an independent pharmacist. Participants, clinicians, outcome assessors, and laboratory personnel remained blinded to treatment allocation throughout the trials. Baseline demographic and clinical characteristics are summarized in Table 1.

### 2.3. Interventions and Study Visits

Participants in the active group received one capsule per day containing ≥5 × 10^9^ colony-forming units (CFU) of *H. coagulans* IDCC 1201 (COA 1201) for 8 weeks (Table 2). Placebo capsules matched the active product in appearance and composition, excluding viable organisms. Both investigational products were manufactured under Good Manufacturing Practice (GMP) standards and stored at 2–8 °C. Viable counts were confirmed both at production and expiration using standard plate count assays on De Man–Rogosa–Sharpe (MRS) agar.

Study visits were conducted at baseline (week 0), week 4, and week 8. The 8-week intervention period was determined based on previous clinical studies evaluating probiotic supplementation. Study visits were scheduled to assess baseline status, monitor participants’ condition and safety during the intervention period, and evaluate final efficacy and safety outcomes after 8 weeks of supplementation.

Adherence was monitored by capsule counts, with compliance defined as ≥80% of expected intake. Concomitant medications, dietary supplements, and adverse events were recorded at each visit. Participants were instructed to avoid initiating antibiotics, probiotics, prebiotics, or fermented products and to maintain their usual diet and lifestyle throughout the study.

Participant compliance was monitored by counting the remaining capsules returned at subsequent study visits. Compliance was calculated as the proportion of capsules consumed relative to the total number of capsules expected to be consumed during the study period. Participants with compliance of 80% or higher were considered to have adhered to the study protocol.

Participants were instructed to maintain their usual dietary habits throughout the intervention period. Dietary assessments were limited to selected dietary habit variables, including the frequency of meat, instant food, and seafood consumption, which were evaluated via questionnaires.

Concomitant medications and medical history were recorded throughout the study period using the case report form. The medications taken by participants were mainly for the management of pre-existing or ongoing medical conditions, including antihypertensive agents, lipid-lowering agents, antidiabetic medications, thyroid hormone replacement therapy, and other medications for chronic or concomitant conditions.

### 2.4. Questionnaires and Clinical Outcomes

#### 2.4.1. Bowel Activity Questionnaire

Participants recorded bowel movement frequency (per week), stool form (Bristol Stool Form Scale, BSFS), defecation duration, incomplete evacuation, abdominal pain (before or during defecation), abdominal bloating and gas, and post-defecation discomfort. Clinical assessments were performed at weeks 0, 4, and 8. The questionnaire items were developed with reference to guidelines from the Ministry of Food and Drug Safety (MFDS) and incorporated validated components of bowel habit assessments.

#### 2.4.2. IBS Symptom Severity Score (IBS-SSS)

Abdominal pain severity and frequency, abdominal bloating, dissatisfaction with bowel habits, and impact on daily activities were assessed using 0–10 Visual Analog Scales (VAS) [27].

#### 2.4.3. IBS Quality of Life (IBS-QOL)

The 34-item IBS-QOL questionnaire, which evaluates eight dimensions (Dysphoria, Interference with activity, Body image, Health worry, Food avoidance, Social reaction, Sexual, and Relationships), was administered. Each item was scored on a 5-point scale, and total scores were converted to a 0–100 scale, with higher values indicating better quality of life [28].

### 2.5. Fecal Sample Collection

Fecal samples were obtained at baseline, week 4, and week 8 using the AccuStool Collection Kit (AccuGene, Incheon, Republic of Korea). One tube containing stabilization buffer was designated for microbiome sequencing, and one empty tube was used for metabolomic analyses. Participants stored samples at −20 °C at home and delivered them within 24 h of collection. Upon receipt, all samples were stored at −80 °C until analysis. The interval between defecation and freezing, transport conditions, and any deviations were documented.

### 2.6. Fecal Microbiome Analysis

Fecal samples (250 mg) were processed using the DNeasy PowerSoil Pro Kit (Qiagen, Venlo, The Netherlands) according to the manufacturer’s instructions. DNA concentration and quality were determined using the Quant-IT PicoGreen assay. Full-length 16S rRNA genes (V1–V9) were amplified with barcoded primers 27F (5′-AGRGTTYGATYMTGGCTCAG-3′) and 1492R (5′-RGYTACCTTGTTACGACTT-3′), following the PacBio amplicon workflow. PCR conditions were as follows: 94 °C for 5 min; 25 cycles of 94 °C for 30 s, 53 °C for 30 s, 72 °C for 90 s; and a final extension at 72 °C for 5 min. Amplicons were purified with SMRTbell cleanup beads, quantified by PicoGreen, and assessed on an Agilent TapeStation D5000. Libraries were prepared using the SMRTbell Express Template Prep Kit 3.0 and sequenced on the PacBio Sequel IIe platform (10 h movies; Macrogen Inc., Seoul, Republic of Korea).

HiFi circular consensus sequences (CCS) were generated with SMRT Link software v 13.1. Primer sequences were trimmed with Cutadapt (v3.2), and reads were processed using DADA2 (v1.18.0) for quality filtering, denoising, and chimera removal. Samples with fewer than 5000 reads were excluded from downstream analysis, and low-abundance features (present in <0.1% of samples or with <10 total counts) were filtered out. Taxonomic classification of amplicon sequence variants (ASVs) was performed using the naive Bayesian classifier in DADA2 (confidence threshold = 50%) against the NCBI 16S reference database. Multiple sequence alignment was performed with MAFFT (v7.475), and phylogenetic trees were constructed with FastTreeMP (v2.1.10). Diversity analyses were performed using QIIME (v1.9.0). Alpha diversity was assessed using the Shannon, Simpson, and Chao1 indices. Beta diversity was calculated using the Bray–Curtis distance and visualized by non-metric multidimensional scaling (NMDS), with group differences evaluated by PERMANOVA (999 permutations). Differential abundance analysis was performed using Analysis of Compositions of Microbiomes with Bias Correction (ANCOM-BC), with *p*-values adjusted using the Benjamini–Hochberg false discovery rate (FDR). LEfSe (Linear Discriminant Analysis Effect Size) was conducted to identify genomic features characterizing the differences between groups. Taxa with an absolute LDA score greater than 2.0 and a *p*-value less than 0.05 (adjusted by FDR) were visualized in a bar chart representing the dominant microbial biomarkers.

### 2.7. Fecal Metabolomics

Fecal extracts were used for untargeted metabolomics profiling. Briefly, 20 mg of fecal sample was homogenized with 100 µL deionized water and 500 µL methanol, vortexed for 10 min, and centrifuged at 13,000 rpm for 5 min at 4 °C. The supernatant was filtered through a 0.2 µm PVDF membrane. A 400 µL aliquot was evaporated to dryness using a vacuum concentrator (VS-802, Visonbionex), and the remaining extract was stored at −80 °C until analysis. Dried extracts were derivatized with 30 µL of methoxyamine hydrochloride (20 mg/mL in pyridine; Sigma, St. Louis, MO, USA) at 30 °C for 90 min, followed by 50 µL of N, O-bis(trimethylsilyl)trifluoroacetamide (BSTFA; Sigma) at 60 °C for 30 min. An alkane mixture served as the retention index standard, and fluoranthene was added as an internal standard. 

Gas chromatography–mass spectrometry (GC–MS) analysis was performed on a Thermo Trace 1310 system (Thermo, Waltham, MA, USA) coupled to a Thermo ISQ LT single quadrupole mass spectrometer and equipped with a DB-5MS column (60 m × 0.25 mm, 0.25 µm film thickness; Agilent, Santa Clara, CA, USA). Derivatized samples were injected at 300 °C in split mode (1:5), with helium as the carrier gas (1.5 mL/min). The oven temperature program was as follows: 50 °C for 2 min; ramp at 5 °C/min to 180 °C (hold for 8 min); 2.5 °C/min to 210 °C; 5 °C/min to 325 °C (hold for 10 min). Electron impact ionization (70 eV) was employed, scanning m/z 35–650 at 5 spectra/s, with the ion source at 275 °C. Spectral processing was performed using Thermo Xcalibur and AMDIS software v2.73 with automated peak detection. Metabolites identification was based on retention indices and spectral matching using the NIST Mass Spectral Search Program (version 2.0, Gaithersburg, MD, USA) and MS-DIAL (http://prime.psc.riken.jp/compms/msdial/main.html; accessed on 11 July 2024). Relative metabolite intensities were determined by normalizing peak areas to the total sum of identified peaks, converted to percentages, and subsequently log-transformed for variance stabilization. Prior to comparative statistical analysis, all data were auto-scaled to standardize the variance and provide each metabolite with equal importance in the statistical models, thereby eliminating scale-dependent bias.

### 2.8. Statistical Analysis

Efficacy analyses were performed in the per-protocol (PP) population, with complementary analyses in the full analysis (FA; modified intention-to-treat) set. Data normality was assessed using the Shapiro–Wilk test. Between-group comparisons of continuous outcomes were analyzed using independent-samples *t*-tests or Mann–Whitney U tests, as appropriate. Within-group changes were assessed using paired *t*-tests or Wilcoxon signed-rank tests to compare the specific metabolic shifts at distinct clinical time points and control high inter-individual baseline variability. Categorical variables were compared using chi-square or Fisher’s exact tests. A two-sided *p*-value < 0.05 was considered statistically significant. In addition to primary paired comparisons, sensitivity analyses were performed using linear mixed-effects models (LMM) with random intercepts for participants to evaluate longitudinal trajectory changes across 0, 4, and 8 weeks (within the COA group) and to assess treatment-by-time interactions (COA vs. placebo). These mixed-effects analyses were conducted using GraphPad Prism (version 11.0; GraphPad Software, San Diego, CA, USA).

For microbiome and metabolomics datasets, multiple comparisons were controlled using the Benjamini–Hochberg FDR method. Multivariate metabolomics analyses, including partial least-squares discriminant analysis (PLS-DA), were performed using MetaboAnalyst 6.0 (https://www.metaboanalyst.ca/, accessed on 11 July 2024). To improve data quality, metabolites with an interquartile range (IQR) within the lower 10% were filtered out.

Statistical analyses were performed using GraphPad Prism (version 11.0) and R (v4.3.2). Differences among experimental groups were analyzed using one-way or two-way ANOVA followed by Tukey’s post-hoc test for multiple comparisons. Data are expressed as mean ± SEM, and statistical significance was defined as *p* < 0.05. 

Safety evaluations were performed in the safety population, defined as all participants who received at least one dose of the study product. Adverse events (AEs) and serious adverse events (SAEs) were summarized by frequency, severity, and relationship to the study product.

## 3. Results

### 3.1. Effects of COA 1201 on Gastrointestinal Symptoms and Quality of Life

A randomized, double-blind, and placebo-controlled clinical trial was conducted to investigate the effects of COA 1201 in adults diagnosed with FBD according to the Rome IV criteria. A total of 38 participants were screened, 19 participants were allocated to the COA 1201 group and 19 to the placebo group, with all completing study procedures (Table 1). Four participants in the placebo group discontinued due to protocol violations (e.g., concomitant use of prohibited medication) or withdrawal of consent. 

Baseline demographic and clinical characteristics showed no statistically significant differences between the groups in mean age, sex distribution, body mass index, lifestyle factors, vital signs, or baseline gastrointestinal symptoms (Table 1). Compliance rates exceeded 94% across visits in both groups, and no participants were excluded for poor adherence (<80% at two consecutive visits). No statistically significant differences in the evaluated dietary habit variables were observed between the placebo and COA groups.

**Figure 1:**
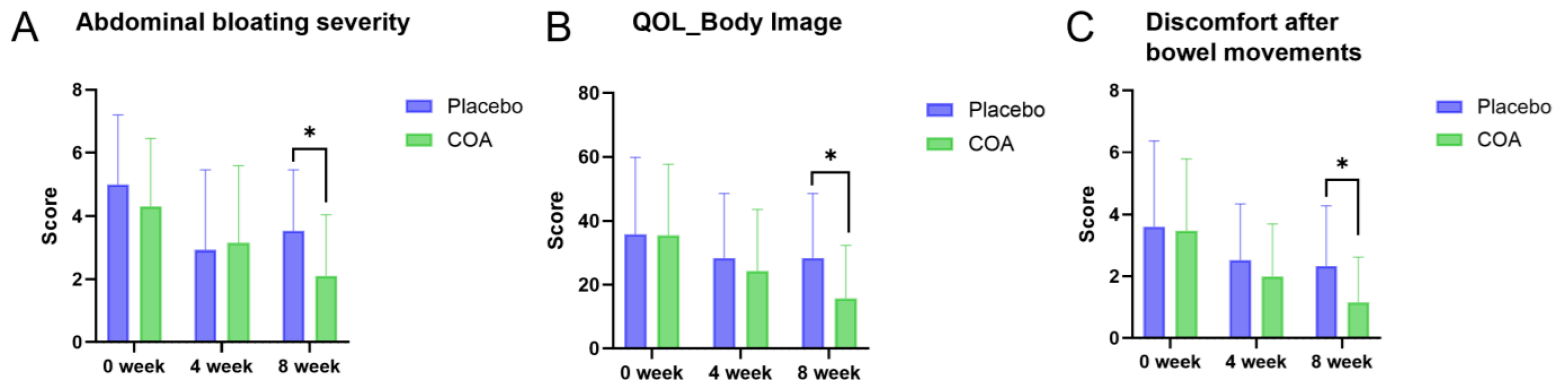
Assessment of bowel activities in participants receiving COA 1201. (**A**) Abdominal bloating severity. (**B**) QOL body image. (**C**) Discomfort after bowel movements. Data are presented as mean ± standard deviation. Significant differences compared to baseline (week 0) are indicated as * *p* < 0.05 using repeated-measures ANOVA with Tukey’s post-hoc analysis.

Changes in gastrointestinal symptoms were assessed using the IBS-SSS, IBS-QOL, and bowel activity questionnaires at 0, 4, and 8 weeks (Section 2.4); all corresponding data are presented in Supplementary Tables S1–S6. From baseline (week 0) to week 8, both groups exhibited reductions in IBS-SSS total scores. In the COA 1201 group, significant within-group improvements were observed across all IBS-SSS domains—abdominal pain severity, abdominal pain frequency, abdominal bloating, dissatisfaction with bowel habits, interference with daily life, and the total scores (all *p* ≤ 0.013, Wilcoxon’s signed rank test). In contrast, improvements in the placebo group were largely confined to the first 4 weeks, with limited changes thereafter. By week 8, the only significant between-group difference was in abdominal bloating severity (*p* = 0.036) (Figure 1A). 

Quality of life, including dysphoria, interpersonal relations, body image, and health worry were increased within COA group. Although total IBS-QOL scores (excluding sexual concerns) increased more in the COA 1201 group than in the placebo group (*p* ≤ 0.002), only the Body Image domain showed a statistically significant difference between-group difference (*p* = 0.047) (Figure 1B). This domain reflects discomfort related to abdominal distension (e.g., feeling or looking bloated, clothing fit, self-perceived appearance), suggesting a clinically relevant improvement in bloating-associated concerns among COA 1201 recipients.

Bowel activity assessments—including bowel movements, fecal volume, stool form, abdominal pain, and bloating—were examined using a Visual Analog Scale (VAS). Several bowel activity indicators, such as number of times of abdominal pains during bowel movements, amount of gas, and discomfort after bowel movements, demonstrated significant differences across the time intervals within COA group, showing meaningful changes between 0 vs. 4 weeks, 4 vs. 8 weeks, and 0 vs. 8 weeks simultaneously. However, only discomfort after bowel movements was significantly reduced at week 8 in the COA 1201 group compared with the placebo group (*p* = 0.043) (Figure 1C). Other differences did not reach statistical significance.

### 3.2. Fecal Microbiota Profiles Following COA 1201 Administration

Metagenome profiling of fecal microbiota was performed to evaluate whether COA 1201 contributed to clinical improvements in FBD. A total of 69 fecal samples were collected at 0, 4, and 8 weeks of intervention, yielding 4,142,270 high-quality HiFi reads (mean 39,450 ± 9175 reads per sample). 

Alpha diversity indices (Chao1, Shannon, and Simpson) did not differ significantly between groups at any time point (Figure S2A). Beta diversity based on Bray–Curtis dissimilarity also showed overlapping microbial community structures between groups, with no significant separation (Figure S2B). 

**Figure 2:**
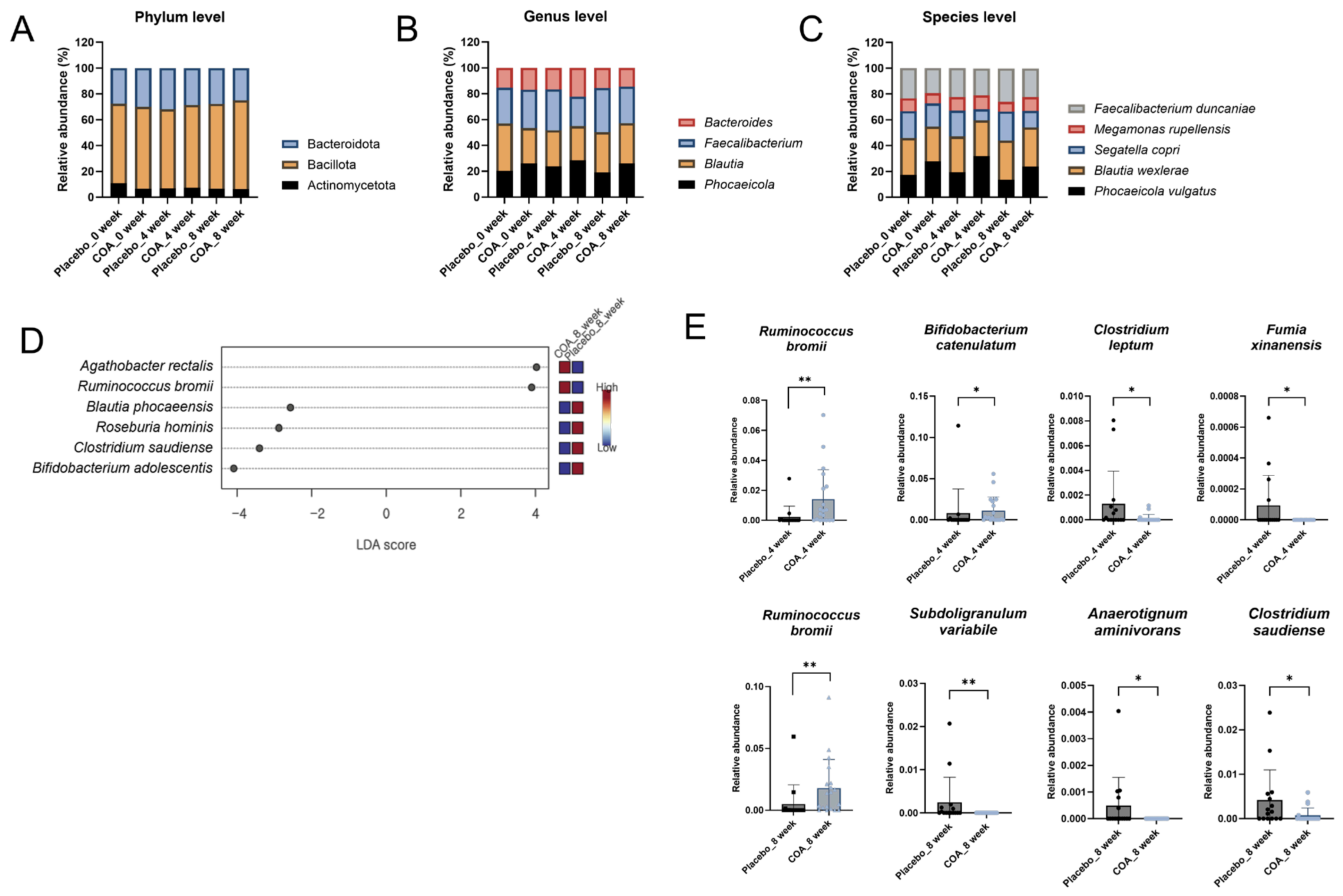
Predominantly characterized fecal microbiota profiles: (**A**) Phylum, (**B**) genus, and (**C**) species-level enrichment analysis and (**D**) linear discriminant effect size (LEfSe) between placebo and COA 1201 groups. (**E**) Differentially abundant species at 4 and 8 weeks in placebo and COA 1201 groups. Significant differences between groups are indicated as * *p* < 0.05 and ** *p* < 0.01 using the Mann–Whitney U-test.

**Figure 3:**
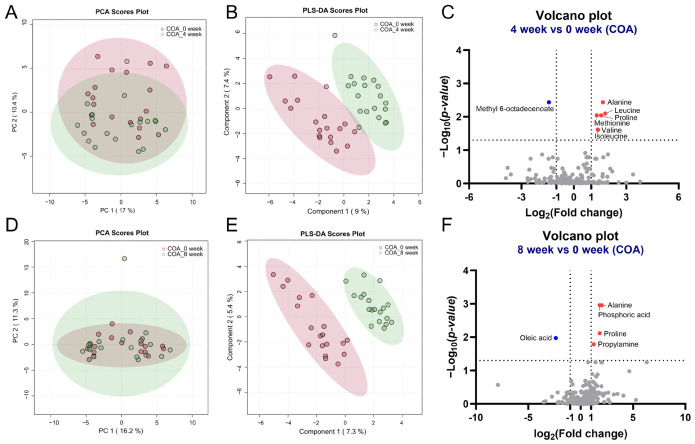
Multivariate and univariate analyses of the fecal metabolome during COA 1201 intervention. Principal component analysis (PCA) score plots comparing (**A**) 0 vs. 4 weeks and (**D**) 0 vs. 8 weeks. Supervised partial least squares discriminant analysis (PLS-DA) score plots comparing (**B**) 0 vs. 4 weeks and (**E**) 0 vs. 8 weeks (auto-scaling). Volcano plots showing differentially abundant metabolites between (**C**) 0 vs. 4 weeks and (**F**) 0 vs. 8 weeks. Red and blue dots represent significantly increased and decreased metabolites, respectively (*p* < 0.05). Gray dots indicate metabolites with no significant change. Significant metabolites were identified based on Student’s *t*-test (*p* < 0.05) and |log2 fold change| > 1.

Despite the absence of global diversity shifts, taxon-level analyses revealed notable compositional changes associated with COA 1201 administration. A total of 9 phyla, 100 genera, and 172 species were identified from the 38 fecal samples (Figure S3). The microbial community was predominantly characterized by the phyla Bacillota, Bacteroidota, and Actinomycetota (Figure 2A), with *Phocaeicola*, *Blautia*, *Faecalibacterium*, and *Bacteroides* being the major genera (Figure 2B). At the species level, *Phocaeicola vulgatus*, *Blautia wexlerae*, *Segatella copri*, *Megamonas rupellensis*, and *Faecalibacterium duncaniae* were most predominant (Figure 2C). Notably, *B. wexlerae* and *F. duncaniae* tended to increase in the COA 1201 group at week 8, suggesting a positive modulation of community balance [29,30]. Linear discriminant analysis effect size (LEfSe) analysis identified taxa that were differentially enriched between the COA 1201 and placebo groups (Figure 2D). Higher linear discriminant analysis (LDA) scores indicate greater effect sizes [31]. At week 8, *Agathobacter rectalis* was the most enriched species in the COA 1201 group (Figure 3E), whereas *Blautia phocaeensis* was most enriched in the placebo group.

At week 4, the relative abundances of *Ruminococcus bromii* and *Bifidobacterium* were significantly increased in the COA 1201 group compared with the placebo group. These differences persisted at week 8, alongside enrichment of other saccharolytic and butyrate-producing species (*A. rectalis*, *R. bromii*) (Figure 2E). In contrast, *Clostridium saudiense*, a potentially pro-inflammatory species, was more abundant in the placebo group. These findings suggest that COA 1201 selectively modulated microbial populations linked to carbohydrate utilization and SCFA production, rather than inducing broad diversity shifts.

### 3.3. Modulation of Fecal Metabolite Profiles by Administration of COA 1201

**Figure 4:**
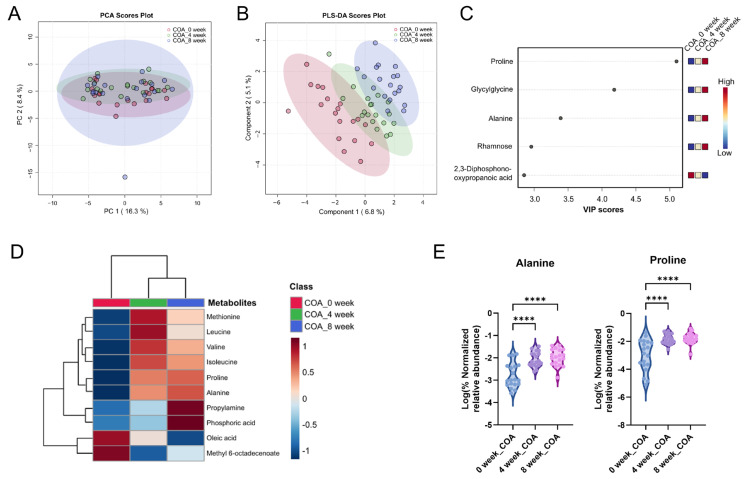
Comparative profiling of the fecal metabolome during COA 1201 intervention. (**A**) PCA score plots at 0, 4, and 8 weeks. (**B**) Supervised PLS-DA score plots at 0, 4, and 8 weeks (auto-scaling). (**C**) Variable Importance in Projection (VIP) scores identifying discriminant metabolites; the top five metabolites with VIP > 2.5 are shown. (**D**) Hierarchical clustering analysis (HCA) across 0, 4, and 8 weeks. (**E**) Normalized relative abundance (%) of exploratory metabolites. Significant metabolites were identified based on Student’s *t*-test (*p* < 0.05), VIP > 2.5, and |log_2_ fold change| > 1. Significant differences between groups are indicated as **** *p* < 0.0001.

**Figure 5:**
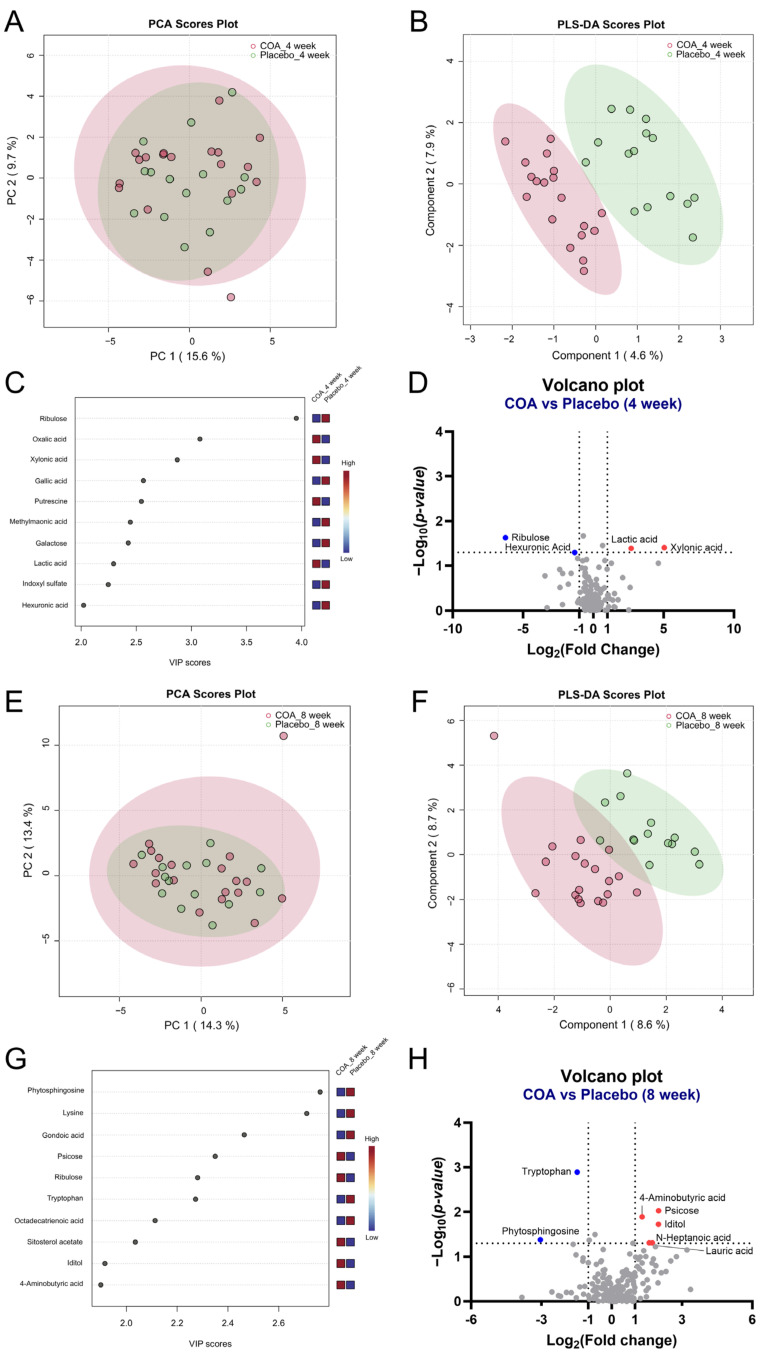
Multivariate and univariate analyses of the fecal metabolome comparing the COA 1201 and placebo groups. PCA score plots at (**A**) 4 weeks and (**E**) 8 weeks. Supervised PLS-DA score plots at (**B**) 4 weeks and (**F**) 8 weeks (auto-scaling). VIP scores identifying discriminant metabolites at (**C**) 4 weeks and (**G**) 8 weeks; the top five metabolites with VIP > 1.5 are shown. Volcano plots showing differentially abundant metabolites at (**D**) 4 weeks and (**H**) 8 weeks. Red and blue dots represent significantly increased and decreased metabolites, respectively (*p* < 0.05). Gray dots indicate metabolites with no significant change. Significant metabolites were identified based on Student’s *t*-test (*p* < 0.05) and |log2 fold change| > 1.

**Figure 6:**
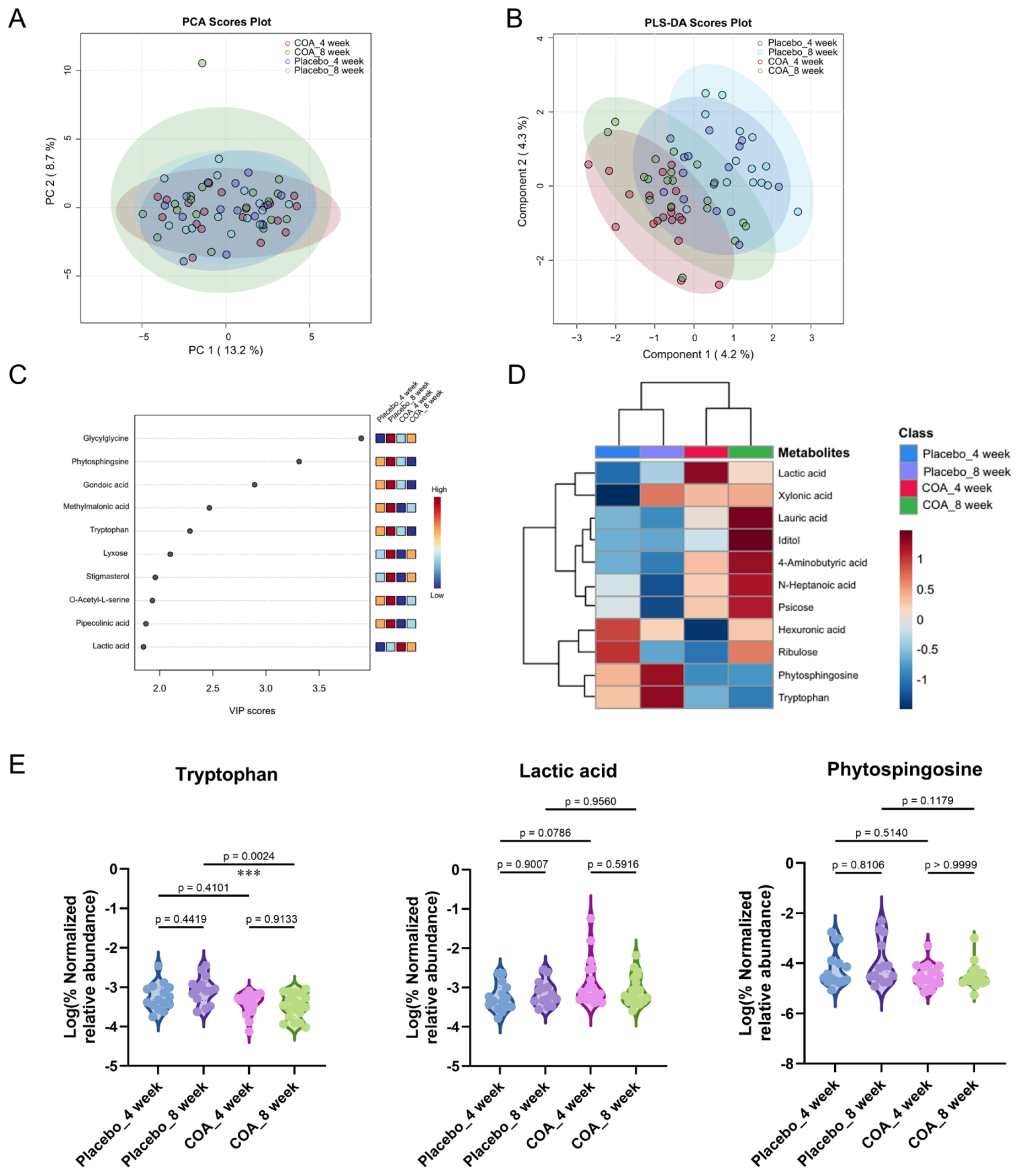
Comparative profiling of the fecal metabolome in the COA 1201 and placebo groups over time. (**A**) PCA score plots at 0, 4, and 8 weeks. (**B**) Supervised PLS-DA score plots at 0, 4, and 8 weeks (auto-scaling). (**C**) VIP scores identifying discriminant metabolites; the top five metabolites with VIP > 1.5 are shown. (**D**) HCA comparing the COA 1201 and placebo groups across 0, 4, and 8 weeks. (**E**) Normalized relative abundance (%) of exploratory metabolites. Significant metabolites were identified based on Student’s *t*-test (*p* < 0.05), VIP > 1.5, and |log_2_ fold change| > 1. Significant differences between groups are indicated as *** *p* < 0.001.

To elucidate the metabolic mechanisms underlying the clinical benefits of COA 1201, untargeted fecal metabolomic profiling was performed using GC–MS (Figure 3, Figure 4, Figure 5 and Figure 6). After rigorous data processing, 312 metabolites were identified for the time-course evaluation of the COA 1201 intervention, and 191 metabolites were identified for the group-wise comparison between the COA 1201 and placebo groups (Table S1). This discrepancy resulted from independent data filtering optimized for statistical models. Multivariate and univariate analyses were conducted to examine temporal changes in metabolite profiles during COA 1201 treatment (Figure 3).

Unsupervised principal component analysis (PCA) revealed overlapping metabolic profiles due to inter-individual variability, explaining ~27% of variance at weeks 0, 4, and 8 (Figure 3A,D). At weeks 0 and 4, PC1 and PC2 accounted for 27.4% of variance, showing a narrow distribution, while weeks 0 and 8 explained 27.5% with similar overlap. These findings reflect the inherent variability of human clinical trials, which can obscure treatment-specific effects [32]. To overcome these limitations and maximize class-specific variance, supervised PLS-DA was applied (Figure 3B,E), revealing a time-dependent expansion in metabolic variance and clearer separation between baseline and week 8 compared with baseline and week 4. 

Subsequent univariate analysis identified exploratory metabolites that differed significantly across COA 1201 intervention periods (Figure 3C,F). Volcano plots (FDR *p* < 0.05, |log_2_ fold change| > 1) showed that six amino acids—alanine, leucine, proline, methionine, valine, and isoleucine—were significantly elevated at week 4. At week 8, alanine and proline remained elevated, along with phosphoric acid and propylamine. In contrast, lipid-related metabolites such as methyl 6-octadecenate and oleic acid decreased significantly at weeks 4 and 8, respectively. These findings indicate that COA 1201 primarily modulated amino acid metabolism, with additional effects on energy and lipid metabolism.

Beyond pairwise comparisons, a comprehensive analysis across weeks 0, 4, and 8 revealed progressive shifts in the fecal metabolome (Figure 4). PCA again showed no clear group separation (Figure 4A), but supervised PLS-DA demonstrated a distinct linear trajectory of metabolic change, accounting for 11.2% of variance over the 8-week intervention (Figure 4B). Key contributors included proline, glycylglycine, alanine, rhamnose, and 2,3-diphosphonooxypropanoic acid (VIP > 2.5) (Figure 4C). With the exception of 2,3-diphosphonooxypropanoic acid, the other four metabolites increased progressively during the COA 1201 intervention. Hierarchical clustering analysis (HCA) confirmed increased amino acids and metabolic byproducts following COA 1201 treatment, alongside decreased lipid metabolites (Figure 4D). 

Methionine and branched-chain amino acids (BCAA: leucine, isoleucine, valine) were early responders, elevated at week 4 but reduced by week 8. In contrast, non-essential amino acids like alanine and proline increased continuously, reaching 3.15-fold and 4.03-fold higher levels for alanine, and 2.94-fold and 3.54-fold higher levels for proline at weeks 4 and 8, respectively (*p* < 0.05) (Figure 4E). These metabolites were consistently identified across volcano plots and PLS-DA, supporting their role as potential biomarkers of COA 1201 intervention.

To evaluate longitudinal metabolic alterations within the COA 1201 intervention group, a sensitivity analysis using a linear mixed-effects model (LMM) was conducted across the 0, 4, and 8 week time points in the COA group. The LMM analysis confirmed statistically significant longitudinal changes in key amino acid metabolites over time, including alanine (*p* = 0.0002) and proline (*p* = 0.0063) (Table S8).

Collectively, COA 1201 modulated amino acid metabolism in a time-dependent manner. The treatment initially promoted microbial BCAA biosynthesis at week 4, followed by sustained increases in non-essential amino acids such as alanine and proline by week 8. These temporal differences likely reflect distinct origins—whether endogenously synthesized by the host or derived from microbial biosynthesis and dietary sources. Overall, COA 1201 induced progressive metabolic changes that support gut health in patients with FBD. 

Comparative metabolomic analysis was conducted to identify the distinct metabolic signatures induced by COA 1201 (Figure 5). Consistent with prior multivariate analyses, PCA did not show clear group separation due to inter-individual variability (Figure 5A,E). In contrast, supervised PLS-DA effectively discriminated between groups, revealing distinct separation at weeks 4 and 8 (Figure 5B,F), accounting for 12.5% and 17.3% of variance, respectively. 

At week 4, although no significant metabolites were identified under the FDR-adjusted *p*-value < 0.05, ribulose, xylonic acid, lactic acid, and hexuronic acid were identified as exploratory metabolites (VIP > 1.5, Raw *p* < 0.05, |log_2_ fold change| > 1) (Figure 5C,D,G,H). Reductions in sugar intermediates (ribulose, hexuronic acid) and increases in fermentation-derived organic acids (xylonic acid, lactic acid) suggest modulation of carbohydrate metabolism. At week 8, phytosphingosine, psicose, tryptophan, iditol, and 4-aminobutyric acid (GABA) were identified, indicating a progression toward bioactive metabolite synthesis. 

Consequently, administration of COA 1201 to FBD patients resulted in a phased progression of the gut metabolome. The early stage (week 4) was characterized by modulation of carbohydrate fermentation-related metabolites, whereas the later stage (week 8) was marked by the synthesis of bioactive compounds such as GABA, which may contribute to a healthier gut environment. 

To further characterize these changes, fecal metabolomic profiling was compared between the COA 1201 and placebo groups at weeks 4 and 8 (Figure 6A,B). Despite overlapping PCA patterns that explained 21.9% of the variance, supervised PLS-DA revealed subtle but significant group differences, accounting for 8.5% of the total variance. Importantly, metabolic profiles were more effectively segregated by COA 1201 intervention than by time point, underscoring treatment-specific effects. Although the multivariate scores showed modest inter-group separation, the model identified metabolites with high VIP scores (>1.5), suggesting that COA 1201 induced distinct shifts in the gut metabolome of FBD patients. 

The top ten metabolites with VIP scores > 1.5 (Figure 6C) included amino acids and derivatives, fatty acids and lipids, organic acids, and carbohydrates. Most amino acids and derivatives—such as tryptophan, O-acetyl-L-serine, pipecolinic acid, and glycylglycine—were reduced in the COA 1201 group compared with placebo, with differences more pronounced at week 8, reflecting a sustained response. These reductions have been associated with the mitigation of intermediate metabolite accumulation linked to microbial imbalance and inflammation [33,34,35,36]. Fatty acids and lipids, including gondoic acid and phytosphingosine, also decreased in abundance, potentially serving as substrates for beneficial microbial proliferation [37,38]. In contrast, lactic acid increased, and methylmalonic acid decreased, suggesting improved fermentation by beneficial bacteria and enhanced metabolic efficiency in the propionate pathway [39,40].

Two metabolites—stigmasterol and lyxose—showed time-dependent changes independent of treatment, increasing at week 8 compared with week 4. Previous studies have reported that these metabolites have conflicting roles: lyxose has been associated with hypertension risk [41], whereas stigmasterol has been shown to improve intestinal barrier function [42]. Despite their high VIP scores, these metabolites did not reach statistical significance in the pairwise volcano plots (Figure 5D,H) and were excluded from further discussion. 

Heatmap analysis (Figure 6D) highlighted six metabolites—lactic acid, GABA, N-heptanoic acid, psicose, phytosphingosine, and tryptophan—as significantly distinct between groups. Specifically, lactic acid, GABA, N-heptanoic acid, and psicose exhibited consistently elevated levels, while phytosphingosine and tryptophan showed reduced levels in the COA 1201 group. Metabolites meeting all three criteria (VIP > 1.5, *p* < 0.05, |log_2_ fold change| > 1) included tryptophan, lactic acid, and phytosphingosine (Figure 6E). Of these, only tryptophan showed a statistically significant reduction at week 8 (*p* = 0.0024). Lactic acid showed greater differences in relative abundance between the COA 1201 and placebo groups at week 4 (*p* = 0.0786) than at week 8. Phytosphingosine was reduced in the COA 1201 group compared with the placebo group at week 8. Although the mean differences between groups were not statistically significant, these metabolites contributed substantially to the separation observed between the COA 1201 and placebo groups.

Furthermore, to evaluate treatment-by-time dynamics between the COA 1201 and placebo group, a linear mixed-effects sensitivity analysis was performed on exploratory metabolites over 8 weeks (Table S10). Notably, lactic acid exhibited a statistically significant Time × Group interaction (F(2, 95) = 4.144, *p* = 0.019), demonstrating a distinct longitudinal response profile in the COA group compared with the placebo group. In contrast, tryptophan showed a significant overall effect of time (F(1, 95) = 7.222, *p* = 0.009) without a significant interaction (*p* = 0.683), while phytosphingosine maintained overall stability across groups and time points (*p* > 0.05).

Collectively, longitudinal and comparative analyses demonstrate that COA 1201 administration modulates fecal metabolite profiles in FBD patients. The intervention promoted carbohydrate fermentation and bioactive metabolite synthesis, increased the levels of lactic acid and GABA, and decreased the levels of tryptophan and lipid metabolites, supporting a healthier gut metabolic environment.

## 4. Discussion

*Heyndrickxia coagulans* IDCC 1201 (COA 1201) has previously been shown to exert anti-inflammatory effects by inhibiting the production of inflammatory mediators and inducible enzymes [20]. In this study, a randomized, double-blind, placebo-controlled trial was conducted in patients with FBD to evaluate the therapeutic effects of COA 1201 administered orally at 5 × 10^9^ CFU per day. After 8 weeks, the COA 1201 group demonstrated greater improvements in abdominal bloating severity (IBS-SSS) and body image scores (IBS-QOL) compared with placebo. Microbiome analysis revealed modest changes in overall bacterial diversity, but significant enrichment of specific taxa, including *A. rectalis* and *R. bromii*. Metabolomic profiling identified alanine, proline, and tryptophan as exploratory metabolites altered by COA 1201. Together, these microbiota–metabolite features correlated with clinical outcomes, suggesting that COA 1201 may promote the growth of beneficial bacteria and the biosynthesis of functional metabolites, thereby supporting intestinal health. 

The placebo group included 7 participants with constipation or IBS-C and 8 participants with diarrhea or IBS-D, whereas the intervention group included 14 participants with constipation or IBS-C and 5 participants with diarrhea or IBS-D. Randomization was not stratified according to FBD subtype, and a numerical imbalance in subtype distribution was observed between the groups. Because the number of participants in each subtype was limited and the study was not powered for subtype-specific efficacy evaluation, formal subgroup analyses were not performed.

Previous studies have reported the clinical benefits of *H. coagulans* strains in alleviating FBD-related symptoms such as intestinal pain, diarrhea, and bloating [43,44,45,46]. Consistent with these reports, our findings showed that COA 1201 reduced abdominal bloating severity and discomfort after bowel movements. Abdominal bloating—characterized by pressure, fullness, and gas—is one of the most common FBD symptoms, affecting ~20% of adults and up to 70% of FBD patients [47,48]. Because bloating negatively impacts quality of life, reducing the severity of abdominal bloating is a clinically meaningful outcome [49]. One of the causes of abdominal bloating is distension during bowel movements [50]; thus, COA 1201 may alleviate bloating by reducing distension-related discomfort. Furthermore, body image dissatisfaction is frequently associated with depression in IBD patients [51,52], underscoring the relevance of the improvements observed in our study. 

Gut dysbiosis is a major contributor to FBD. Consistent with prior reports, patients with diarrhea often exhibit reduced levels of commensal bacteria such as *Ruminococcus* spp. and *Bifidobacterium* spp., alongside higher levels of pathogenic taxa such as *Clostridium* spp. [53]. Among our findings, *R. bromii* was particularly notable, as its relative abundance was significantly higher in the COA 1201 group at weeks 4 and 8 (Figure 2E). *Ruminococcus bromii* is a prevalent gut bacterium capable of degrading resistant starch [54,55], producing SCFAs such as butyrate, acetate, and formate [56]. Notably, SCFAs are critical for colonocyte health [57,58], suggesting that enrichment of *R. bromii* by COA 1201 may contribute to improved gut function. In addition, a previous study has shown that *H. coagulans* promotes the growth of *Ruminococcus* spp. [59].

*Agathobacter rectalis* (formerly known as *Eubacterium rectale*) is one of the bacteria most closely associated with butyrate production in the colon [60,61]. Several health benefits of *A. rectalis* have been reported, including anti-inflammatory effects and the strengthening of the gut mucosal barrier [62,63,64]. Interestingly, the concurrent enrichment of *R. bromii* and *A. rectalis* in the COA 1201 group suggests a synergistic metabolic benefits for carbohydrate fermentation and essential substance production for SCFAs production [65,66].

*Bifidobacterium catenulatum* has been reported to be significantly reduced in fecal samples from colorectal cancer (CRC) patients compared with healthy controls [67]. In our study, the relative abundance of *B. catenulatum* increased in the COA 1201 group at week 4, supporting an improvement in gut dysbiosis. In contrast, the relative abundance of *C. leptum* and *C. saudiense* decreased in the COA 1201 group compared with placebo at weeks 4 and 8, respectively (Figure 3B). 

*Clostridium leptum* is one of the most abundant commensal bacteria, comprising 16–25% of adult fecal microbiota, and its presence is closely linked to microbial balance in the gut [68]. Although many studies have reported reduced levels of *C. leptum* in patients with IBD [69,70,71,72], its precise role in gut health remains unclear and warrants further investigation. 

In addition to *C. leptum*, *C. saudiense* has been reported as a commensal bacterium. However, recent evidence suggests potential pathogenicity, including a case of hepatocellular carcinoma associated with *C. saudiense* infection [73]. Moreover, *C. saudiense* has been identified as one of the original sources of tetracycline resistance genes [74]. Therefore, the observed decline in the abundance of *C. saudiense* following COA 1201 treatment may help mitigate risks related to pathogenicity and antibiotic resistance. 

Another species reduced by COA 1201 treatment was *S. variabile*. This bacterium has been reported to be highly abundant in the gut microbiota of children with food sensitization [75] and to contribute to the development of autoantibodies associated with rheumatoid arthritis [76]. Conversely, some studies have described *S. variabile* as a butyrate-producing commensal bacterium in the human gut [77], underscoring the need for further research to clarify its role in human health. 

Research on *Fumia xinanensis* and *Anaerotignum aminivorans* remains limited. Notably, *F. xinanensis* has been shown to increase in response to a high-fat diet and decrease following the administration of feruloyl acetone, a compound with immunomodulatory effects [78,79]. Thus, the reduction of *F. xinanensis* observed with COA 1201 treatment may indicate a beneficial impact on gut health. 

Metabolomic analysis revealed significant differences across time following COA 1201 treatment, as well as between the COA 1201 and placebo groups (Figure 3, Figure 4, Figure 5 and Figure 6), under the multi-thresholds (*p*-value < 0.05, |log2 Fold change| > 1, and VIP score > 1.5) [80]. Notably, alanine and proline were exploratory metabolites contributing to the temporal metabolic shift, while tryptophan was identified as a treatment-specific metabolite unique to COA 1201. Previous studies have shown that other *H. coagulans* strains also regulate amino acid–related metabolites, improving gut recovery [81] and alleviating gut inflammation by modulating amino acid, sugar, and lipid metabolism [82].

Alanine, a non-essential amino acid synthesized by the human body, consists of a pyruvate backbone with an attached amino (-NH_2_) group [83]. Since SCFAs such as acetate and propionate can be produced via alanine fermentation in the gut [84], elevated alanine levels may promote intestinal homeostasis by lowering luminal pH and enhancing energy supply to colonocytes [85]. Consistent with this, *H. coagulans* has been shown to upregulate amino acid metabolism, including alanine, while reducing *Clostridium* spp. and alleviating diarrhea [86].

Proline metabolism plays a critical role in maintaining gut microbiome homeostasis and strengthening intestinal barrier function [87,88,89]. Positive correlations have been reported between proline levels and the abundance of beneficial bacteria such as *Lactobacillus* spp., *Bifidobacterium* spp., and *Bacteroides* spp. [90,91]. Beyond these effects, proline systemically influences the metabolism of other amino acids, including BCAAs, thereby promoting SCFA production, modulating colonocyte signaling, and exerting immunomodulatory effects [92]. Given that *H. coagulans* possesses the proline iminopeptidase (PIP) gene, which enables the cleavage of the N-terminal proline residue from peptides [93], the free proline released by COA 1201 may contribute to restoring intestinal homeostasis. Since proline depletion has been observed in imbalanced gut environments such as DSS-induced colitis and IBD, proline supplementation has been used to alleviate bowel disorders [94,95]. Supporting this, a recent study demonstrated that a high abundance of *R. bromii* was positively associated with proline metabolism in healthy children [96]. In line with these findings, the elevated levels of alanine and proline observed after COA 1201 intervention may promote the growth and fermentation of beneficial microbiota, ultimately contributing to a reduction in abdominal bloating and discomfort after bowel movements. 

Methionine and BCAAs (valine, leucine, and isoleucine) also emerged as early responders to COA 1201 treatment. These amino acids are essential for microbial proliferation and intestinal barrier integrity [97,98]. While the role of *H. coagulans* in modulating methionine and BCAA levels has primarily been studied in the context of enhancing dietary protein utilization and muscle protein synthesis [99,100,101,102], our findings suggest that their modulation within the gut environment may also contribute to improved quality of life. Specifically, the observed improvement in Body Image scores, despite no significant weight loss, may be linked to increased BCAA availability. Further research is needed to clarify these mechanisms. These findings suggest that the proteolytic and metabolic capabilities of COA 1201 are consistently manifested in the intestinal environment, enhancing amino acid availability to support gut barrier integrity and reduce inflammation in patients with FBD [103,104].

Beyond amino acid metabolism, phosphoric acid and propylamine exhibited distinct increases after 8 weeks of COA 1201 administration. Phosphoric acid is a critical substrate in phosphorylation and ATP-dependent pathways of amino acid metabolism [105,106]. Furthermore, the increase in propylamine, a byproduct of microbial amino acid decarboxylation, suggests that COA 1201 enhances amino acid metabolic turnover [107]. Together, these metabolites support the observed metabolic flux involving alanine, proline, methionine, and BCAAs. 

Shifts in lipid metabolism were also evident following COA 1201 treatment, with reductions in two fatty acid metabolites highlighting its multifaceted impact on gut metabolism. Research on long-chain fatty acid esters within the human gut is limited; however, the observed decrease in methyl 6-octadecenoate (also known as methyl petrselinate) following COA 1201 administration may indicate metabolic utilization by the gut microbiota [108,109]. Because these fatty acids serve as essential structural components of microbial membranes, their consumption may reflect enhanced microbial proliferation and improved gut health [110]. In contrast, consistent reductions in oleic acid levels may alleviate metabolic lipotoxicity, which is known to compromise intestinal barrier integrity and promote gut dysbiosis [111]. Overall, COA 1201 intervention appears to reconfigure the gut environment by modulating fatty acid profiles, balancing microbial growth factors with the removal of harmful metabolites to restore intestinal homeostasis.

A comparison with the placebo group revealed that metabolic profiles following COA 1201 treatment exhibited distinct clustering patterns depending on metabolite class. Due to the inter-individual baseline variation typical of human clinical samples, no individual metabolites satisfied the conservative FDR-adjusted *p*-value < 0.05 criteria compared to placebo group. Nevertheless, utilizing a combination of a raw *p*-value < 0.05, |log2 Fold change| > 1, and VIP score > 1.5 allowed us to reliably identify the exploratory metabolites. Exploratory metabolites driving this separation included tryptophan, lactic acid, and phytospingosine. Tryptophan is fermented by gut microbiota such as *Clostridium* spp. and *Bacteroides* spp. [112]. Notably, *C. leptum* has been shown to modulate the tryptophan–indole pathway, thereby inducing inflammation [113,114]. The reduced abundance of *C. leptum* in the COA 1201 group may therefore explain the observed decrease in tryptophan levels. Furthermore, although *R. bromii* encodes tryptophan biosynthetic enzymes [115,116], our data revealed a paradox, with increased *R. bromii* abundance occurring alongside reduced tryptophan levels. This discrepancy may reflect an active metabolic shift rather than simple metabolite accumulation. Importantly, tryptophan metabolism is closely linked to the gut–brain axis [117]. In this study, decreased tryptophan levels coincided with increased GABA, another key neurotransmitter in this axis [118]. Given that *H. coagulans* IDCC 1201 interacts with GABA receptors and exhibits a high GABA conversion rate [23], COA 1201 may exert systemic effects on gut–brain signaling. 

Lactic acid, produced both by host metabolism and gut microbiota, functions as an energy source and metabolic substrate [119]. Elevated lactic acid levels can protect the gut barrier by promoting epithelial cell migration and inhibiting colitis development [120]. Consistent with this, the increased lactic acid levels observed in the COA 1201 group may contribute to protection against gastrointestinal inflammation such as IBD or FBD [121]. Given that *H. coagulans* is widely used as a starter culture for lactic acid production [122,123,124], these findings align with its metabolic characteristics. The increased abundance of *R. bromii* following COA 1201 treatment also supports this observation, as *R. bromii* produces lactic acid through carbohydrate degradation [125]. Although ANOVA did not show statistical significance between groups, lactic acid was identified as a key contributor to group discrimination in multivariate analysis, underscoring its biological relevance. 

Phytosphingosine was another metabolite contributing to group separation. Recent studies have shown that phytosphingosine improves gut and skin inflammation, strengthens barrier function, and modulates the microbiome [37,126,127,128]. As a primary structural component of cellular membranes and a key intermediate in sphingolipid metabolism [129], phytosphingosine enhances tight junction protein expression, including ZO-1, occludin, and claudin-3, thereby improving barrier integrity [130]. Interestingly, phytosphingosine levels were higher in the placebo group. Although differences were not statistically significant, this finding suggests that COA 1201 may influence intestinal lipid balance to maintain homeostasis. Given that excessive accumulation of structural lipids has been linked to cellular stress and apoptosis [131], the observed phytosphingosine profiles may reflect similar processes. Further studies are needed to clarify these mechanisms.

## 5. Limitations

This study identified significant microbiota and metabolome features following COA 1201 treatment in patients with FBDs. Because FBDs encompass broad conditions such as IBS, FC, and FD, clinical outcome–specific metabolic profiles and treatment responses remain poorly understood. While these findings require validation through clinical correlations and mechanistic studies—specifically evaluating parameters like SCFAs, intestinal permeability, inflammatory cytokines, immune cell activation, and epithelial function—this study is the first to characterize fecal metabolite changes in FBD patient populations. 

Specifically, we applied multi-thresholds (*p*-value < 0.05, |log2 Fold change| > 1, and VIP score > 1.5) to minimize potential statistical errors and false-positive discoveries when screening exploratory metabolites within this 38-participant cohort. Nevertheless, given the relatively small sample size and the use of unadjusted *p*-values, these identified exploratory metabolites should be interpreted as candidate biomarkers, highlighting the need for validation in larger, independent cohorts in future trials. Also, since detailed dietary assessments—such as food frequency questionnaires, precise dietary records, or comprehensive nutrient intake analyses—were not conducted, the potential contribution of unmeasured dietary factors to the observed fecal metabolite changes cannot be entirely ruled out. Future studies incorporating comprehensive dietary tracking are required to clearly distinguish intervention-driven metabolic shifts from diet-associated variations. 

## 6. Conclusions

In this randomized, double-blind, placebo-controlled trial, *H. coagulans* IDCC 1201 (COA 1201) was associated with clinically meaningful improvements in FBD symptoms. COA 1201 notably reduced abdominal bloating severity and discomfort after bowel movements and improved the Body Image domain of IBS-QOL after 8 weeks. Although overall α- and β-diversity remained comparable between groups, taxon-level shifts favored saccharolytic and SCFA-producing microbes, including *R. bromii*, during COA 1201 administration. Untargeted metabolomics revealed enrichment of functional metabolites such as alanine and proline, alongside distinct patterns in tryptophan, lactic acid, and phytosphingosine that clearly separated COA 1201 from placebo profiles. In conclusion, this study provides the first comprehensive analysis of fecal metabolites in FBD patients, demonstrating that COA 1201 can improve clinical symptoms by modulating both the gut microbiome and metabolome.

## Figures and Tables

**Table 1:** Baseline characteristics of patients. Values are expressed as mean ± standard deviation unless otherwise indicated. *p*-values were obtained using the Mann–Whitney U-test between groups.

Variables	Placebo	COA	*p*-Value
Sex (*n*, %)			0.484
Male	7 (46.7)	6 (31.6)	
Female	8 (53.3)	13 (68.4)	
Age (years)	42.80 ± 13.97	36.79 ± 7.65	0.349
Height (cm)	165.38 ± 6.62	166.33 ± 8.16	0.681
Weight (kg)	69.95 ± 15.78	67.82 ± 11.78	0.857
BMI (kg/m^2^)	25.72 ± 4.70	24.39 ± 3.01	0.566
Smoking (*n*, %)			0.492
Yes	0 (0.0)	2 (10.53)	
No	15 (100)	16 (84.21)	
Ex-smoker	0 (0.0)	1 (5.26)	
Alcohol drinking (*n*, %)			0.314
Yes	5 (33.3)	10 (52.6)	
No	10 (66.7)	9 (47.4)	
Light-intensity physical activity	1.80 ± 1.08	1.95 ± 1.93	0.765
Moderate-intensity physical activity	1.33 ± 1.29	1.42 ± 1.30	0.861
Sleep duration (h/day)	6.57 ± 1.24	6.68 ± 1.24	0.690
Systolic blood pressure (mm Hg)	124.33 ± 13.98	125.16 ± 10.25	0.871
Diastolic blood pressure (mm Hg)	71.93 ± 13.92	73.79 ± 11.23	0.751
Pulse (beats/min)	80.00 ± 10.46	76.39 ± 10.15	0.375
Compliance (%) at week 4	96.22 ± 4.50	94.46 ± 5.99	0.383
Compliance (%) at week 8	95.40 ± 5.61	95.86 ± 4.48	0.936

Values are expressed as mean ± standard deviation unless otherwise indicated. *p*-values were obtained using the Mann–Whitney U-test between groups.

**Table 2:** Capsule components for participants.

	Component	Purpose	Formulation Ratio (%)
Primary ingredient	*H. coagulans* IDCC 1201 live bacteria	Probiotics	20
Supplementary ingredients	Corn starch	Carrier/Diluent	38.5
	Maltodextrin	Cryoprotectant/Stabilizer	38.5
	Silicon dioxide	Anti-caking agent/Glidant	2.0
	Magnesium stearate	Lubricant	1.0
Total			100

## Data Availability

The data presented in this study are available from the corresponding author upon reasonable request.

## Supplementary Materials

The following supporting information can be downloaded at: https://www.mdpi.com/article/10.3390/nu18152466/s1, Figure S1: CONSORT diagram; Figure S2: (A) Alpha-diversity indices (Chao1, Shannon, Simpson) of fecal microbiota in placebo and COA 1201 groups at 0, 4, and 8 weeks, analyzed using Mann–Whitney U-test. (B) Beta-diversity based on non-metric multidimensional scaling (NMDS) and Bray–Curtis dissimilarity index of fecal microbiota in placebo and COA 1201 groups at 0, 4, and 8 weeks.; Figure S3: (A) Phylum, (B) genus, and (C) species-level enrichment analysis between placebo and COA groups. Table S1: Comparison of bowel symptom severity between groups (Placebo vs. COA group); Table S2: Comparison of bowel symptom severity within COA groups across different time points (0 vs. 4, 4 vs. 8, and 0 vs. 8 week); Table S3: Comparison of quality of life between groups (Placebo vs. COA group); Table S4: Comparison of quality of life within COA groups across different time points (0 vs. 4, 4 vs. 8, and 0 vs. 8 week); Table S5: Comparison of bowel activity between groups (Placebo vs COA group); Table S6: Comparison of bowel activity within COA groups across different time points (0 vs. 4, 4 vs. 8, and 0 vs. 8 week); Table S7: A total of 313 metabolites identified in the time-course evaluation of the COA 1201 intervention.; Table S8: Sensitivity analysis of key metabolites in the time-course evaluation of the COA 1201 intervention using linear mixed-effects models (LMM).; Table S9: A total of 191 metabolites identified in the group-wise comparison between the COA 1201 and placebo groups.; Table S10: Sensitivity analysis of key metabolites in the group-wise comparison between the COA 1201 and placebo groups using linear mixed-effects models (LMM).

## Abbreviations

The following abbreviations are used in this manuscript:

FBD	Functional bowel disorders
COA	*Heyndrickxia coagulans*
IBS	Irritable bowel syndrome
SSS	Symptom Severity Score
QOL	Quality of Life
FC	Functional constipation
FD	Functional diarrhea
FAB	Functional abdominal bloating
FAD	Functional abdominal distension
SCFAs	Short-chain fatty acids
ICH-GCP	Declaration of Helsinki and International Conference on Harmonization Good Practice
CRIS	Clinical Research Information Service
GI	Gastrointestinal
CFU	Colony-forming units
GMP	Good Manufacturing Practice
MRS	De Man–Rogosa–Sharpe
MFDS	Ministry of Food and Drug Safety
VAS	Visual Analog Scales
CCS	Circular consensus sequences
LEfSe	Linear Discriminant Analysis Effect Size
ANCOM-BC	Analysis of Compositions of Microbiomes with Bias Correction
NMDS	Non-metric multidimensional scaling
FDR	False discovery rate
BSTFA	O-bis(trimethylsilyl)trifluoroacetamide
GC-MS	Gas chromatography–mass spectrometry
PP	Per-protocol
FA	Full analysis
PLS-DA	Partial least-squares discriminant analysis
PCA	Principal component analysis
IQR	Interquartile range
AEs	Adverse events
SAEs	Serious adverse events
HCA	Hierarchical clustering analysis
BCAAs	Branched-chain amino acids
GABA	4-aminobutyric acid
VIP	Variable importance in projection
ANOVA	Analysis of variance
PIP	Proline iminopeptidase
