# Supplementary material for: Heyndrickxia coagulans IDCC 1201 Alters Gut Microbiome and Metabolome in Patients with Functional Bowel Disorders
Source: Nutrients. 2026 Jul 29;18(15):2466. doi: 10.3390/nu18152466 (PMC13467927; doi:10.3390/nu18152466)
Supplement: Supplementary file 1 [file nutrients-18-02466-s001.zip › nutrients-4375161-supplementary.pdf]

Table S1. Comparison of bowel symptom severity between groups (Placebo vs COA group).

	0 week			4 week			8 week		
	Placebo	COA	*P*-value††	Placebo	COA	*P*-value†	Placebo	COA	*P*-value†
Abdominal pain severity	4.40 ± 2.03	4.53 ± 2.41	0.762	3.00 ± 2.07	2.84 ± 2.29	0.787	2.47 ± 1.60	3.05 ± 2.39	0.493
Abdominal pain frequency	2.87 ± 1.64	3.37 ± 2.41	0.720	1.60 ± 1.06	2.37 ± 2.43	0.480	1.93 ± 1.10	2.11 ± 2.45	0.568
Abdominal bloating severity	5.00 ± 2.20	4.32 ± 2.14	0.362	2.93 ± 2.52	3.16 ± 2.43	0.869	3.53 ± 1.92	2.11 ± 1.94	0.036*
Dissatisfaction of bowel habits	5.07 ± 2.31	5.74 ± 1.76	0.330	4.33 ± 1.40	4.63 ± 1.54	0.566	4.13 ± 1.55	3.74 ± 1.66	0.568
Interference with quality of life	4.27 ± 2.52	4.84 ± 1.83	0.345	3.47 ± 1.77	3.37 ± 2.29	0.964	3.80 ± 1.82	2.74 ± 1.85	0.134
Total	21.60 ± 9.23	22.79 ± 7.92	0.738	15.33 ± 5.80	16.37 ± 9.31	0.898	15.87 ± 6.35	13.74 ± 8.74	0.376

Values are expressed as means ± standard deviation.

†Significant differences between groups at each week were analyzed using the Mann–Whitney U test (*$p < 0.05$, **$p < 0.01$, and ***$p < 0.001$).

Table S2. Comparison of bowel symptom severity within COA groups across different time points (0 vs 4, 4 vs 8, and 0 vs 8 week).

(Variables	COA group								
	0 week	4 week	*P*-value†	4 week	8 week	*P*-value†	0 week	8 week	*P*-value†
Abdominal pain severity	4.53 ± 2.41	2.84 ± 2.29	0.001**	2.84 ± 2.29	3.05 ± 2.39	0.517	4.53 ± 2.41	3.05 ± 2.39	0.013*
Abdominal pain frequency	3.37 ± 2.41	2.37 ± 2.43	0.010**	2.37 ± 2.43	2.11 ± 2.45	0.258	3.37 ± 2.41	2.11 ± 2.45	0.002**
Abdominal bloating severity	4.32 ± 2.14	3.16 ± 2.43	0.061	3.16 ± 2.43	2.11 ± 1.94	0.045*	4.32 ± 2.14	2.11 ± 1.94	<0.001***
Dissatisfaction of bowel habits	5.74 ± 1.76	4.63 ± 1.54	0.007**	4.63 ± 1.54	3.74 ± 1.66	0.002**	5.74 ± 1.76	3.74 ± 1.66	<0.001***
Interference with quality of life	4.84 ± 1.83	3.37 ± 2.29	0.003**	3.37 ± 2.29	2.74 ± 1.85	0.192	4.84 ± 1.83	2.74 ± 1.85	<0.001***
Total	22.79 ± 7.92	16.37 ± 9.31	<0.001***	16.37 ± 9.31	13.74 ± 8.74	0.068	22.79 ± 7.92	13.74 ± 8.74	<0.001***

Table S3. Comparison of quality of life between groups (Placebo vs COA group).

	0 week			4 week			8 week		
	Placebo	COA	*P*-value†	Placebo	COA	*P*-value†	Placebo	COA	*P*-value†
Dysphoria	25.93 ± 19.74	20.47 ± 15.33	0.465	18.52 ± 19.46	12.58 ± 14.27	0.341	17.05 ± 16.89	7.17 ± 9.27	0.080
Interference with Activity	28.67 ± 23.26	27.63 ± 22.07	0.897	19.00 ± 19.38	19.47 ± 18.48	0.869	18.00 ± 20.16	14.74 ± 12.64	0.951
Interpersonal relations	18.51 ± 16.32	16.08 ± 13.31	0.816	14.27 ± 15.43	10.53 ± 10.62	0.622	11.48 ± 13.56	7.02 ± 7.98	0.705
Food Avoidance	41.10 ± 21.47	40.79 ± 28.17	0.993	28.88 ± 15.39	26.32 ± 21.39	0.494	23.89 ± 17.22	19.74 ± 15.77	0.442
Social Reaction	26.67 ± 18.82	36.84 ± 25.16	0.245	18.33 ± 19.97	21.71 ± 21.59	0.667	18.33 ± 19.97	17.11 ± 17.78	0.850
Sexual concerns	14.17 ± 18.22	5.26 ± 12.72	0.095	10.00 ± 14.33	3.29 ± 7.02	0.153	8.33 ± 16.14	1.97 ± 6.27	0.254
Body Image	35.83 ± 24.03	35.53 ± 22.15	0.958	28.33 ± 20.30	24.34 ± 19.31	0.564	28.33 ± 20.30	15.79 ± 16.58	0.047*
Health Worry	39.17 ± 18.82	48.03 ± 29.24	0.334	25.83 ± 19.75	25.00 ± 22.05	0.758	21.67 ± 13.75	16.45 ± 19.57	0.165

Table S4. Comparison of quality of life within COA groups across different time points (0 vs 4, 4 vs 8, and 0 vs 8 week).

Variables	COA group								
	0 week	4 week	*P*-value†	4 week	8 week	*P*-value†	0 week	8 week	*P*-value†
Dysphoria	20.47 ± 15.33	12.58 ± 14.27	0.006**	12.58 ± 14.27	7.17 ± 9.27	0.004**	20.47 ± 15.33	7.17 ± 9.27	<0.001***
Interference with Activity	27.63 ± 22.07	19.47 ± 18.48	0.006**	19.47 ± 18.48	14.74 ± 12.64	0.100	27.63 ± 22.07	14.74 ± 12.64	0.001**
Interpersonal relations	16.08 ± 13.31	10.53 ± 10.62	0.006**	10.53 ± 10.62	7.02 ± 7.98	0.045*	16.08 ± 13.31	7.02 ± 7.98	<0.001***
Food Avoidance	40.79 ± 28.17	26.32 ± 21.39	0.013*	26.32 ± 21.39	19.74 ± 15.77	0.166	40.79 ± 28.17	19.74 ± 15.77	0.002**
Social Reaction	36.84 ± 25.16	21.71 ± 21.59	0.001**	21.71 ± 21.59	17.11 ± 17.78	0.223	36.84 ± 25.16	17.11 ± 17.78	<0.001***
Sexual concerns	5.26 ± 12.72	3.29 ± 7.02	0.688	3.29 ± 7.02	1.97 ± 6.27	0.625	5.26 ± 12.72	1.97 ± 6.27	0.250
Body Image	35.53 ± 22.15	24.34 ± 19.31	0.015*	24.34 ± 19.31	15.79 ± 16.58	0.012*	35.53 ± 22.15	15.79 ± 16.58	<0.001***
Health Worry	48.03 ± 29.24	25.00 ± 22.05	0.004**	25.00 ± 22.05	16.45 ± 19.57	0.031*	48.03 ± 29.24	16.45 ± 19.57	<0.001***

Table S5. Comparison of bowel activity between groups (Placebo vs COA group).

	0 week			4 week			8 week		
	Placebo	COA	*P*-value†	Placebo	COA	*P*-value†	Placebo	COA	*P*-value†
Number of bowel movement per week	6.00 ± 3.72	6.42 ± 7.35	0.342	6.27 ± 3.22	5.74 ± 4.58	0.266	6.20 ± 3.26	5.32 ± 3.73	0.259
Number of times of irritant bowel movements	3.93 ± 2.37	5.16 ± 2.52	0.116	2.93 ± 1.71	2.89 ± 2.05	0.855	3.13 ± 1.85	3.05 ± 2.37	0.747
Number of times when bowel movements felt incomplete	3.93 ± 2.19	4.37 ± 2.34	0.645	3.13 ± 1.77	2.26 ± 1.63	0.190	3.20 ± 1.97	2.26 ± 2.23	0.092
Number of times of abdominal pains before bowel movements	3.33 ± 2.09	5.32 ± 2.96	0.040	2.27 ± 1.39	3.58 ± 2.59	0.148	2.47 ± 1.06	2.95 ± 2.86	0.815
Number of times of abdominal pains during bowel movements	3.07 ± 1.79	4.00 ± 2.83	0.470	1.93 ± 1.28	2.95 ± 2.72	0.457	2.07 ± 1.10	2.37 ± 2.59	0.831
Degree of abdominal pain	3.53 ± 2.00	4.42 ± 2.32	0.276	2.47 ± 1.13	2.89 ± 2.21	0.744	2.27 ± 1.03	2.79 ± 2.23	0.449
Amount of gas	5.47 ± 2.72	6.58 ± 2.46	0.262	4.53 ± 2.70	4.58 ± 2.69	0.966	3.60 ± 2.35	3.16 ± 2.67	0.569
Discomfort after bowel movements	3.60 ± 2.77	3.47 ± 2.32	0.910	2.53 ± 1.81	2.00 ± 1.70	0.421	**2.33 ± 1.95**	**1.16 ± 1.46**	**0.043***
Discomfort caused by constipation	2.80 ± 2.43	3.21 ± 2.46	0.571	2.40 ± 2.03	2.05 ± 1.68	0.825	1.93 ± 2.09	1.53 ± 1.81	0.635

Table S6. Comparison of bowel activity within COA groups across different time points (0 vs 4, 4 vs 8, and 0 vs 8 week).

Variables	COA group								
	0 week	4 week	*P*-value†	4 week	8 week	*P*-value†	0 week	8 week	*P*-value†
Number of bowel movement per week	6.42 ± 7.35	5.74 ± 4.58	0.923	5.74 ± 4.58	5.32 ± 3.73	0.425	6.42 ± 7.35	5.32 ± 3.73	0.551
Defecation time	2.00 ± 0.75	1.79 ± 0.71	0.388	1.79 ± 0.71	1.68 ± 0.75	0.727	2.00 ± 0.75	1.68 ± 0.75	0.109
Amount of feces	2.47 ± 0.84	2.63 ± 0.83	0.508	2.63 ± 0.83	2.53 ± 0.90	0.781	2.47 ± 0.84	2.53 ± 0.90	>0.999
Number of times of irritant bowel movements	5.16 ± 2.52	2.89 ± 2.05	<0.001***	2.89 ± 2.05	3.05 ± 2.37	0.987	5.16 ± 2.52	3.05 ± 2.37	0.015*
Number of times when bowel movements felt incomplete	4.37 ± 2.34	2.26 ± 1.63	0.001**	2.26 ± 1.63	2.26 ± 2.23	0.899	4.37 ± 2.34	2.26 ± 2.23	0.002**
Shape of the feces	3.58 ± 1.54	3.89 ± 0.88	0.339	3.89 ± 0.88	3.84 ± 0.76	>0.999	3.58 ± 1.54	3.84 ± 0.76	0.547
Number of times of abdominal pains before bowel movements	5.32 ± 2.96	3.58 ± 2.59	<0.001***	3.58 ± 2.59	2.95 ± 2.86	0.089	5.32 ± 2.96	2.95 ± 2.86	0.001**
Number of times of abdominal pains during bowel movements	4.00 ± 2.83	2.95 ± 2.72	0.015*	2.95 ± 2.72	2.37 ± 2.59	0.016*	4.00 ± 2.83	2.37 ± 2.59	0.002**
Degree of abdominal pain	4.42 ± 2.32	2.89 ± 2.21	0.004**	2.89 ± 2.21	2.79 ± 2.23	0.869	4.42 ± 2.32	2.79 ± 2.23	0.005**
Amount of gas	6.58 ± 2.46	4.58 ± 2.69	0.008**	4.58 ± 2.69	3.16 ± 2.67	0.043*	6.58 ± 2.46	3.16 ± 2.67	<0.001***
Discomfort after bowel movements	3.47 ± 2.32	2.00 ± 1.70	0.001**	2.00 ± 1.70	1.16 ± 1.46	0.016*	3.47 ± 2.32	1.16 ± 1.46	<0.001***
Discomfort caused by constipation	3.21 ± 2.46	2.05 ± 1.68	0.063	2.05 ± 1.68	1.53 ± 1.81	0.210	3.21 ± 2.46	1.53 ± 1.81	0.003**

Table S7. A total of 313 metabolites identified in the time-course evaluation of the COA 1201 intervention.

	COA 1201 (4 week vs 0 week)			COA 1201 (8 week vs 0 week)			ANOVA_COA 1201 (0, 4, and 8 week)			
Metabolites	Log_2 (FC)	p value	FDR adjusted p value	Log_2 (FC)	p value	FDR adjusted p value	f value	p value	FDR adjusted p value	VIP score
(2-Aminoethyl) phosphonic acid	-0.297	0.448	0.914	1.103	0.648	0.903	0.676	0.513	0.883	0.274
1,2,4-Butanetriol	-2.643	0.368	0.889	-1.695	0.724	0.916	0.907	0.410	0.883	0.296
1,2-Benzenediol	1.059	0.679	0.943	5.269	0.273	0.717	0.844	0.436	0.883	0.990
1,2-Propanediol	-2.230	0.164	0.687	-1.656	0.768	0.928	1.379	0.261	0.754	0.191
1,3-Diaminopropane	-0.230	0.715	0.948	0.268	0.506	0.877	0.296	0.745	0.947	0.425
1,3-Dihydroxyacetone	0.057	0.259	0.825	1.107	0.078	0.454	2.199	0.121	0.533	0.854
1,3-Dihydroxypropan-2-one dimer	0.399	0.089	0.544	1.135	0.013	0.227	3.914	0.026	0.266	1.073
1,5-Anhydrosorbitol	-0.255	0.997	0.997	-1.295	0.110	0.496	1.991	0.146	0.579	1.729
13-Methyltetradecanoic acid	0.214	0.731	0.948	0.005	0.738	0.916	0.082	0.921	0.980	0.290
16-Hydroxypalmitic acid	-1.304	0.111	0.626	-0.609	0.194	0.658	1.260	0.292	0.782	1.472
18-Methylnonadecanoic acid	-0.191	0.203	0.737	0.778	0.919	0.982	0.815	0.448	0.883	0.073
1-Aminocyclopropane-1-carboxylic acid	1.642	0.116	0.626	2.147	0.133	0.555	1.429	0.248	0.738	1.688
1-Monopalmitin	-0.664	0.144	0.682	-0.753	0.067	0.446	2.442	0.097	0.478	0.623
2,3-Butanediol	-0.687	0.099	0.581	0.566	0.161	0.599	1.340	0.270	0.757	1.553
2,3-Dihydroxy-2-methyl-propanoic acid	-0.232	0.225	0.773	-0.273	0.389	0.839	0.830	0.442	0.883	0.660
2,3-Dihydroxybutyric acid	-1.211	0.009	0.171	1.127	0.446	0.865	2.257	0.114	0.525	0.449
2,3-Diphosphonooxypropanoic acid	-2.002	0.219	0.767	-7.901	0.022	0.266	2.151	0.126	0.540	2.885
2,4-Diaminobutyric acid	0.377	0.443	0.914	0.406	0.649	0.903	0.283	0.754	0.947	0.412
2-Aminobutyric acid	1.690	0.009	0.171	2.038	0.002	0.063	6.242	0.004	0.087	2.817
2'-Deoxyadenosine	-0.438	0.258	0.825	-1.777	0.358	0.835	0.844	0.435	0.883	0.790
2'-Deoxyinosine	0.030	0.411	0.906	1.564	0.643	0.903	0.282	0.755	0.947	0.626

2'-Deoxyuridine	0.021	0.793	0.949	0.978	0.124	0.530	1.511	0.230	0.729	0.886
2-Hydroxy-2-methylbutyric acid	-0.235	0.829	0.962	2.032	0.359	0.835	0.877	0.422	0.883	0.634
2-Hydroxybutyric acid	-0.199	0.605	0.920	-0.498	0.082	0.455	1.911	0.158	0.608	0.678
2-Hydroxycaproic acid	0.917	0.522	0.920	1.511	0.715	0.916	0.150	0.861	0.977	0.265
2-Hydroxycinnamic acid	-2.464	0.192	0.707	-1.762	0.804	0.942	1.015	0.369	0.883	0.281
2-Hydroxyisobutyric acid	-0.886	0.184	0.701	-1.325	0.076	0.454	1.882	0.162	0.610	0.959
2-Hydroxypalmitic acid	0.220	0.751	0.948	0.274	0.728	0.916	0.075	0.928	0.980	0.199
2-Isopropylmalic acid	0.413	0.797	0.949	1.115	0.205	0.674	0.840	0.437	0.883	0.731
2-Ketoglutaric acid	1.133	0.370	0.889	1.523	0.107	0.496	1.254	0.293	0.782	0.991
2-Methylglutaric acid	-0.210	0.545	0.920	0.556	0.780	0.929	0.408	0.667	0.947	0.212
2-Methylserine	1.499	0.038	0.357	2.337	0.012	0.227	3.564	0.035	0.332	1.729
2-Monoolein	1.926	0.413	0.906	0.277	0.902	0.974	0.461	0.633	0.947	0.096
2-Monopalmitin	-0.269	0.631	0.923	-0.214	0.512	0.877	0.282	0.755	0.947	0.202
2-Penten-1-ol	-0.498	0.414	0.906	1.407	0.730	0.916	0.223	0.801	0.955	0.311
2-Piperidone	0.808	0.613	0.920	1.596	0.092	0.478	1.787	0.177	0.636	1.227
2-Pyridone	-0.290	0.315	0.848	-0.053	0.626	0.902	0.385	0.682	0.947	0.187
2-Pyrrolidinone	-0.089	0.907	0.965	1.024	0.669	0.912	0.144	0.866	0.977	0.269
3-(3-Hydroxyphenyl)propanoic acid	0.393	0.859	0.965	0.582	0.783	0.929	0.038	0.963	0.994	0.382
3,4-Dihydroxybenzeneacetic acid	1.463	0.313	0.848	-0.102	0.677	0.914	0.588	0.559	0.921	0.343
3,4-Dihydroxybutyric acid	-0.106	0.601	0.920	1.278	0.037	0.352	4.123	0.022	0.250	1.445
3,6-Anhydro-galactose	-0.706	0.157	0.682	-0.403	0.691	0.916	1.345	0.269	0.757	0.160
3-Aminoisobutanoic acid	0.141	0.615	0.920	0.718	0.002	0.063	5.025	0.010	0.164	2.287
3-Aminopentanedioic acid	2.204	0.053	0.423	1.185	0.066	0.446	2.508	0.091	0.468	1.374
3-Deoxy-galactose	-0.941	0.724	0.948	0.638	0.654	0.903	0.337	0.715	0.947	0.432
3-Hydroxy-3-methylglutaric acid	0.335	0.629	0.923	1.419	0.019	0.266	2.852	0.066	0.439	1.377
3-Hydroxybenzoic acid	-0.059	0.332	0.869	1.085	0.046	0.407	3.126	0.052	0.415	1.036

3-Hydroxybutyric acid	0.258	0.883	0.965	-0.189	0.330	0.820	0.750	0.477	0.883	0.815
3-Hydroxyisovaleric acid	-0.012	0.952	0.980	0.895	0.425	0.846	0.585	0.561	0.921	0.325
3-Hydroxyphenylacetic acid	0.311	0.899	0.965	0.655	0.593	0.902	0.249	0.781	0.955	0.664
3-Indoleacetic acid	-0.548	0.308	0.848	-0.567	0.610	0.902	0.554	0.578	0.939	0.468
3-Ketoadipate	-0.761	0.747	0.948	-0.440	0.776	0.928	0.185	0.832	0.965	0.255
3-Methyl-2-oxopentanoate	0.845	0.131	0.669	0.657	0.182	0.643	1.460	0.241	0.729	0.762
3-Methylaspartic acid	0.746	0.055	0.430	1.982	0.029	0.299	3.088	0.054	0.419	1.859
3-O-Octadecylglycerol	-0.472	0.363	0.889	1.387	0.797	0.942	0.320	0.727	0.947	0.184
4',6,7-Trimethoxyflavonol	-1.948	0.535	0.920	-1.041	0.614	0.902	0.239	0.788	0.955	0.594
4-Aminobenzoic acid	0.479	0.675	0.943	1.225	0.370	0.839	0.430	0.653	0.947	0.541
4-Aminobutanoic acid	-0.192	0.922	0.966	-0.076	0.876	0.971	0.014	0.986	0.997	0.130
4-Aminobutyric acid	-0.287	0.663	0.943	-0.032	0.599	0.902	0.500	0.609	0.946	0.334
4-Hydroxybenzaldehyde	0.866	0.036	0.357	2.011	0.104	0.496	2.169	0.124	0.538	1.076
4-Hydroxybenzoic acid	-0.022	0.977	0.992	0.807	0.185	0.643	1.200	0.309	0.795	0.811
4-Hydroxybutyric acid	0.001	0.770	0.948	0.599	0.487	0.877	0.305	0.738	0.947	0.310
4-Hydroxymandelic acid	0.129	0.619	0.920	1.170	0.663	0.912	0.134	0.875	0.977	0.316
4-Hydroxyphenyl ethanol	0.104	0.460	0.914	2.643	0.155	0.597	2.444	0.096	0.478	1.159
4-Hydroxyphenylacetic acid	-1.317	0.230	0.773	-1.579	0.185	0.643	1.209	0.306	0.795	1.222
4-Methylcatechol	0.411	0.186	0.701	1.417	0.001	0.063	6.367	0.003	0.086	1.291
4-Pyridoxic acid	-2.651	0.153	0.682	-2.739	0.152	0.593	1.675	0.197	0.683	1.092
5,6-Dimethylbenzimidazole	2.116	0.141	0.682	2.065	0.063	0.446	1.823	0.171	0.635	1.569
5-Aminovaleric acid	0.170	0.909	0.965	0.302	0.835	0.954	0.022	0.978	0.997	0.205
5-Hydroxymethyluracil	4.618	0.575	0.920	5.001	0.022	0.266	3.418	0.040	0.348	2.240
5-Ketogluconic acid	0.329	0.720	0.948	-0.117	0.224	0.674	0.609	0.548	0.915	0.679
5-Methylcytosine	-0.222	0.307	0.848	0.118	0.878	0.971	0.850	0.433	0.883	0.058
6-Aminocaproic acid	-0.984	0.406	0.906	-1.205	0.553	0.902	0.397	0.675	0.947	0.684

6-Hydroxynicotinic acid	-0.658	0.781	0.948	-0.599	0.616	0.902	0.136	0.873	0.977	0.485
Acetoacetic acid	0.441	0.738	0.948	-0.189	0.689	0.916	0.282	0.756	0.947	0.264
Acetylserine	-0.249	0.771	0.948	1.607	0.232	0.674	0.890	0.417	0.883	1.270
Aconitic acid	0.278	0.612	0.920	0.880	0.943	0.984	0.195	0.823	0.960	0.063
Adenine	-0.864	0.006	0.157	0.556	0.240	0.674	2.657	0.079	0.450	0.643
Adipic acid	0.272	0.633	0.923	0.500	0.221	0.674	0.772	0.467	0.883	0.651
Alanine	1.657	0.000	0.004	2.011	0.000	0.001	19.913	0.000	0.000	3.429
Alanyl-L-alanine	1.034	0.078	0.523	1.199	0.036	0.348	2.687	0.077	0.448	2.057
Allopyranose	-2.550	0.045	0.381	-2.194	0.065	0.446	2.839	0.067	0.439	2.060
Alpha-Ketobutyrate	3.388	0.075	0.522	3.512	0.101	0.496	1.705	0.191	0.671	1.457
Alpha-Ketobvalerate	-1.816	0.005	0.151	0.260	0.491	0.877	3.889	0.026	0.266	0.486
Aminomalonic acid	0.059	0.366	0.889	0.303	0.274	0.717	0.791	0.458	0.883	0.536
Anthranilic acid	0.301	0.722	0.948	2.516	0.065	0.446	2.506	0.091	0.468	1.341
Arabinitol	-3.613	0.080	0.523	-2.058	0.628	0.902	1.785	0.178	0.636	0.378
Arabinose	-0.194	0.775	0.948	0.741	0.378	0.839	0.764	0.471	0.883	0.628
Arachidic acid	-0.408	0.540	0.920	-0.156	0.386	0.839	0.467	0.630	0.947	0.492
Arachidonic acid	-0.663	0.510	0.920	1.277	0.862	0.971	0.124	0.884	0.977	0.128
Arbutin	-0.393	0.466	0.914	0.710	0.832	0.954	0.237	0.790	0.955	0.156
Asparagine	-1.845	0.750	0.948	-0.921	0.556	0.902	0.500	0.609	0.946	0.423
Aspartic acid	0.445	0.119	0.629	0.794	0.071	0.446	2.108	0.131	0.554	0.911
Azelaic acid	-0.021	0.524	0.920	0.496	0.970	0.994	0.262	0.770	0.955	0.021
Benzoic acid	-0.938	0.012	0.192	-0.396	0.208	0.674	3.174	0.050	0.410	0.551
Benzyl isovalerate	-0.016	0.884	0.965	3.037	0.056	0.446	3.170	0.050	0.410	1.157
Beta-Alanine	-0.751	0.907	0.965	-0.818	0.810	0.943	0.032	0.968	0.994	0.172
Beta-Gentiobiose	-0.161	0.673	0.943	0.323	0.942	0.984	0.128	0.880	0.977	0.056
Beta-sitosterol	-1.300	0.137	0.678	2.245	0.870	0.971	0.820	0.446	0.883	0.130

Butane-1,2,3-triol	-0.970	0.115	0.626	-0.029	0.491	0.877	1.313	0.277	0.759	0.698
Cadaverine	1.426	0.415	0.906	0.112	0.588	0.902	0.375	0.689	0.947	0.699
Caffeic acid	0.514	0.666	0.943	1.273	0.438	0.859	0.327	0.722	0.947	0.521
Cellobiose	-0.289	0.870	0.965	0.130	0.564	0.902	0.337	0.715	0.947	0.342
Chalinasterol	-0.622	0.305	0.848	1.161	0.239	0.674	0.885	0.419	0.883	0.860
Chimyl alcohol	-0.404	0.456	0.914	-0.524	0.586	0.902	0.322	0.726	0.947	0.401
Cholestane	-0.555	0.061	0.455	0.195	0.513	0.877	1.606	0.210	0.706	0.497
Cholesterol	0.024	0.813	0.957	0.821	0.381	0.839	0.490	0.616	0.946	0.497
Citraconic acid	0.679	0.915	0.965	1.122	0.984	0.994	0.009	0.991	0.997	0.026
Citric acid	-0.736	0.451	0.914	0.302	0.991	0.994	0.345	0.710	0.947	0.008
Creatinine	-0.067	0.462	0.914	0.974	0.514	0.877	0.949	0.394	0.883	0.748
Cystamine	-0.038	0.558	0.920	-0.488	0.585	0.902	0.218	0.805	0.955	0.469
Cysteinylglycine	-0.358	0.174	0.687	2.355	0.820	0.947	0.480	0.622	0.946	0.135
Daidzein	0.186	0.595	0.920	0.353	0.408	0.846	0.372	0.691	0.947	1.659
Decanedioic acid	0.720	0.171	0.687	1.890	0.238	0.674	0.915	0.407	0.883	0.640
Decanoic acid	-0.323	0.819	0.961	0.266	0.669	0.912	0.230	0.795	0.955	0.261
Desaminotyrosine	0.113	0.725	0.948	-0.331	0.985	0.994	0.096	0.909	0.978	0.020
Docosahexaenoic acid	-1.674	0.169	0.687	-0.489	0.410	0.846	0.839	0.438	0.883	0.603
Docosanoic acid	-0.191	0.871	0.965	0.258	0.859	0.971	0.058	0.943	0.984	0.090
Docosenoic acid	-2.167	0.088	0.544	-0.483	0.743	0.916	1.360	0.265	0.757	0.229
Dopamine	0.221	0.073	0.521	0.357	0.092	0.478	2.313	0.109	0.506	0.803
Elaidic acid	-0.069	0.784	0.948	-0.983	0.247	0.689	0.678	0.512	0.883	1.003
Erythritol	-3.893	0.151	0.682	-3.354	0.308	0.788	1.326	0.274	0.757	0.810
Erythronic acid	0.558	0.893	0.965	0.816	0.765	0.928	0.089	0.915	0.980	0.201
Erythrose	0.071	0.526	0.920	0.675	0.212	0.674	1.001	0.374	0.883	0.620
Ethanolamine	0.648	0.003	0.136	0.467	0.677	0.914	2.873	0.065	0.439	0.188

Ferulic acid	-0.429	0.989	0.995	0.244	0.266	0.715	0.993	0.377	0.883	0.678
Fructose	-0.953	0.800	0.949	-0.805	0.720	0.916	0.224	0.800	0.955	0.214
Fructose-1-phosphate	-1.178	0.313	0.848	0.355	0.498	0.877	1.505	0.231	0.729	0.436
Fucitol	-1.125	0.040	0.357	-0.751	0.123	0.530	2.340	0.106	0.506	0.835
Fucose	0.150	0.619	0.920	-0.245	0.898	0.974	0.158	0.854	0.977	0.064
Fumaric acid	0.064	0.509	0.920	0.280	0.745	0.916	0.204	0.816	0.960	0.245
Galactinol dihydrate	0.106	0.724	0.948	1.436	0.140	0.573	1.325	0.274	0.757	0.892
Galactitol	-0.437	0.873	0.965	-0.475	0.702	0.916	0.179	0.837	0.967	0.192
Galactonic acid	0.462	0.356	0.889	2.454	0.738	0.916	0.586	0.560	0.921	0.191
Galactose	-1.698	0.375	0.893	-1.766	0.520	0.877	0.515	0.600	0.946	0.636
Galactosylglycerol	-0.415	0.585	0.920	0.707	0.892	0.974	0.115	0.892	0.977	0.078
Galacturonic acid	-0.443	0.596	0.920	0.203	0.959	0.994	0.193	0.825	0.960	0.038
Gallic acid	-0.149	0.308	0.848	0.004	0.336	0.820	0.607	0.549	0.915	0.750
Gentisic acid	-0.647	0.034	0.357	0.515	0.965	0.994	3.024	0.057	0.433	0.025
Gluconic acid	-1.351	0.472	0.914	0.118	0.683	0.916	0.710	0.496	0.883	0.409
Gluconolactone	0.436	0.956	0.981	0.362	0.934	0.982	0.008	0.992	0.997	0.041
Glucosamic acid	-0.309	0.285	0.848	0.507	0.768	0.928	0.871	0.424	0.883	0.146
Glucose	-0.685	0.305	0.848	-0.183	0.909	0.978	0.902	0.412	0.883	0.080
Glucose-1-phosphate	-0.239	0.573	0.920	4.075	0.131	0.554	1.541	0.223	0.729	1.441
Glucose-6-phosphate	0.424	0.615	0.920	-0.078	0.354	0.835	0.348	0.708	0.947	0.530
Glucosone	-0.287	0.442	0.914	0.328	0.975	0.994	0.364	0.697	0.947	0.013
Glutamic acid	0.429	0.478	0.915	0.503	0.382	0.839	0.472	0.626	0.947	0.861
Glutamyl-Valine	-0.309	0.150	0.682	1.879	0.983	0.994	0.539	0.586	0.941	0.011
Glutaric acid	-0.927	0.877	0.965	-0.728	0.645	0.903	0.229	0.796	0.955	0.320
Glyceraldehyde	0.027	0.768	0.948	1.587	0.090	0.478	2.081	0.135	0.560	1.171
Glyceric acid	-0.371	0.569	0.920	0.227	0.744	0.916	0.401	0.672	0.947	0.171

Glycerol	-0.285	0.390	0.906	-0.908	0.012	0.227	3.748	0.030	0.292	1.201
Glycerol 3-phosphate	-0.100	0.368	0.889	0.024	0.443	0.863	0.536	0.588	0.941	0.415
Glycine	0.557	0.022	0.282	0.158	0.684	0.916	2.500	0.092	0.468	0.216
Glycolic acid	-0.234	0.308	0.848	1.476	0.599	0.902	0.773	0.467	0.883	0.237
Glycylglycine	0.372	0.357	0.889	6.317	0.002	0.063	8.702	0.001	0.028	4.229
Gondoic acid	-0.620	0.474	0.914	-1.028	0.368	0.839	0.445	0.643	0.947	0.851
Hexose	1.564	0.749	0.948	-0.364	0.624	0.902	0.101	0.905	0.977	0.354
Hexuronic acid	-0.958	0.490	0.920	0.183	0.402	0.846	1.219	0.304	0.795	0.564
Histidine	1.910	0.740	0.948	-0.948	0.174	0.638	0.769	0.469	0.883	1.324
Homogentisic acid	1.039	0.425	0.914	0.609	0.418	0.846	0.409	0.666	0.947	0.611
Homoserine	0.278	0.206	0.738	0.963	0.031	0.315	2.940	0.061	0.439	0.833
Hydrocaffeic acid	-2.157	0.469	0.914	-0.775	0.612	0.902	0.287	0.752	0.947	0.666
Hydrocinnamic acid	-0.320	0.846	0.965	-0.640	0.823	0.948	0.032	0.969	0.994	0.226
Hydroxyglutaric acid	0.917	0.040	0.357	1.107	0.006	0.143	4.675	0.013	0.190	1.550
Hydroxyphenyllactic acid	0.272	0.913	0.965	-0.579	0.475	0.877	0.386	0.682	0.947	0.613
Hydroxyproline	1.227	0.013	0.192	4.648	0.004	0.117	5.929	0.005	0.101	2.459
Hydroxypropionic acid	-0.768	0.264	0.832	-0.734	0.238	0.674	0.842	0.437	0.883	0.691
Hypotaurine	0.209	0.412	0.906	0.107	0.269	0.717	0.674	0.514	0.883	0.746
Hypoxanthine	-0.971	0.190	0.705	0.757	0.413	0.846	0.788	0.460	0.883	0.709
Iditol	-1.112	0.368	0.889	0.308	0.424	0.846	1.575	0.216	0.718	0.569
Indole-3-lactate	-0.825	0.395	0.906	2.790	0.481	0.877	1.081	0.346	0.865	0.644
Indoxyl sulfate	3.001	0.728	0.948	-1.274	0.277	0.717	0.841	0.437	0.883	0.858
Inosine	-0.388	0.830	0.962	0.616	0.393	0.839	0.694	0.504	0.883	0.555
Inositol	-0.679	0.468	0.914	-1.169	0.058	0.446	2.194	0.121	0.533	0.930
Inositol 4-phosphate	-2.478	0.012	0.192	-1.450	0.158	0.599	4.031	0.023	0.251	0.952
Isoleucine	1.388	0.001	0.027	1.474	0.008	0.168	8.098	0.001	0.035	2.341

Isomaltose	-1.318	0.396	0.906	1.012	0.649	0.903	0.785	0.461	0.883	0.422
Ketoisovaleric acid	1.132	0.093	0.560	2.639	0.080	0.455	1.934	0.154	0.602	1.427
Ketoleucine	-0.676	0.995	0.997	-0.669	0.926	0.982	0.006	0.994	0.997	0.069
Kojic acid	0.347	0.437	0.914	-0.041	0.695	0.916	0.309	0.735	0.947	0.363
Lactic acid	1.702	0.682	0.943	-0.223	0.361	0.835	0.736	0.484	0.883	0.467
Lactitol	-1.407	0.172	0.687	-0.436	0.502	0.877	0.963	0.388	0.883	0.488
Lactobionic acid	-0.385	0.767	0.948	0.041	0.513	0.877	0.483	0.619	0.946	0.418
Lanosterol	0.165	0.879	0.965	0.759	0.278	0.717	0.737	0.483	0.883	0.978
Lauric acid	-1.333	0.345	0.889	-1.289	0.934	0.982	0.721	0.491	0.883	0.063
Leucine	1.790	0.000	0.009	1.310	0.052	0.432	9.300	0.000	0.026	1.986
Leucrose	-0.164	0.729	0.948	1.127	0.350	0.835	0.942	0.396	0.883	0.630
Levodopa	0.658	0.578	0.920	0.979	0.529	0.888	0.222	0.801	0.955	0.420
Levoglucosan	-0.518	0.807	0.954	0.554	0.224	0.674	1.518	0.228	0.729	0.741
Linoleic acid	0.054	0.930	0.968	-0.853	0.147	0.582	1.301	0.281	0.762	1.053
Linolenic acid	0.042	0.983	0.993	-0.133	0.579	0.902	0.218	0.805	0.955	0.422
Lysine	-2.065	0.778	0.948	-3.527	0.392	0.839	0.737	0.483	0.883	1.026
Lyxose	-0.365	0.913	0.965	0.327	0.224	0.674	1.002	0.374	0.883	1.223
Malic acid	0.199	0.230	0.773	0.397	0.423	0.846	0.707	0.498	0.883	0.403
Malonic acid	-0.100	0.939	0.973	0.870	0.501	0.877	0.372	0.691	0.947	0.340
Maltitol	3.796	0.370	0.889	2.318	0.089	0.478	1.034	0.363	0.883	1.112
Maltose	-0.825	0.521	0.920	-0.820	0.420	0.846	0.373	0.691	0.947	0.750
Mannose	-0.119	0.650	0.936	0.084	0.433	0.855	0.382	0.684	0.947	0.498
Margaric acid	0.610	0.509	0.920	0.625	0.895	0.974	0.222	0.802	0.955	0.094
Melibiose	-0.030	0.602	0.920	1.106	0.730	0.916	0.107	0.899	0.977	0.164
Methionine	1.309	0.000	0.010	0.435	0.011	0.227	10.750	0.000	0.012	2.839
Methyl 6-octadecenoate	-1.429	0.000	0.004	0.030	0.052	0.432	7.763	0.001	0.038	0.925

Methyl salicylate	0.044	0.487	0.920	1.567	0.026	0.287	4.051	0.023	0.251	0.997
Methylamine	0.132	0.315	0.848	-0.185	0.229	0.674	2.230	0.117	0.531	0.435
Methyl-galactopyranoside	0.835	0.041	0.357	0.335	0.398	0.844	2.317	0.108	0.506	0.683
Methylglutaric acid	-0.127	0.965	0.986	0.548	0.750	0.917	0.069	0.934	0.980	0.260
Methylmalonic acid	0.430	0.683	0.943	1.458	0.544	0.899	0.510	0.603	0.946	0.729
Methylsuccinic acid	-0.046	0.832	0.962	0.258	0.458	0.877	0.484	0.619	0.946	0.508
Monomyristin	-0.495	0.149	0.682	-0.279	0.161	0.599	1.489	0.235	0.729	0.509
Monoolein	0.300	0.778	0.948	-0.215	0.552	0.902	0.360	0.699	0.947	0.220
Mucic acid	0.854	0.654	0.937	0.492	0.737	0.916	0.108	0.898	0.977	0.328
Myristic acid	0.150	0.775	0.948	0.300	0.735	0.916	0.066	0.936	0.980	0.220
N-Acetylglucosamine	0.271	0.240	0.795	0.346	0.234	0.674	1.377	0.261	0.754	1.421
N-Acetyl-L-aspartic acid	-0.483	0.591	0.920	-0.172	0.801	0.942	0.342	0.712	0.947	0.173
N-Acetyl-L-leucine	0.099	0.892	0.965	2.306	0.104	0.496	2.063	0.137	0.562	1.093
N-Acetyl-mannosamine	0.298	0.500	0.920	0.093	0.538	0.897	0.297	0.744	0.947	0.276
N-Acetylornithine	1.917	0.115	0.626	0.140	0.972	0.994	1.883	0.162	0.610	0.019
N-Acetylputrescine	-0.160	0.511	0.920	1.908	0.145	0.581	2.519	0.090	0.468	0.712
N-Alpha-Acetyl-L-lysine	1.957	0.004	0.149	0.932	0.040	0.370	4.854	0.012	0.171	1.408
N-Heptanoic acid	-1.343	0.251	0.816	-0.030	0.806	0.942	0.672	0.515	0.883	0.151
Nicotinic acid	-0.043	0.440	0.914	0.120	0.495	0.877	0.405	0.669	0.947	0.324
Nonadecylic acid	0.058	0.593	0.920	1.707	0.518	0.877	0.273	0.762	0.952	0.373
Nonanoic acid	-1.163	0.057	0.437	0.109	0.602	0.902	2.006	0.144	0.579	0.420
Norepinephrine	-0.815	0.767	0.948	0.307	0.450	0.867	0.652	0.525	0.890	0.553
Norvaline	2.074	0.036	0.357	1.699	0.069	0.446	2.682	0.078	0.448	1.450
O-Acetyl-L-serine	-0.071	0.764	0.948	0.457	0.497	0.877	0.289	0.750	0.947	0.302
Octadecanol	0.544	0.912	0.965	0.351	0.640	0.903	0.116	0.891	0.977	0.446
Octadecatrienoic acid	0.691	0.845	0.965	-0.385	0.464	0.877	0.447	0.642	0.947	0.598

Octanedioic acid	-0.155	0.556	0.920	2.260	0.335	0.820	1.212	0.305	0.795	0.535
Oleic acid	-0.211	0.160	0.682	-2.369	0.000	0.012	5.889	0.005	0.101	2.373
Ornithine	1.299	0.011	0.192	0.562	0.053	0.432	4.354	0.018	0.222	1.428
Oxalic acid	-0.754	0.763	0.948	-0.318	0.263	0.715	0.679	0.511	0.883	1.244
Palmitic acid	-0.351	0.366	0.889	-0.774	0.019	0.266	3.415	0.040	0.348	1.048
Palmitoleic acid	1.661	0.674	0.943	-0.095	0.935	0.982	0.117	0.890	0.977	0.049
Palmitoleyl alcohol	1.065	0.458	0.914	0.635	0.620	0.902	0.729	0.487	0.883	0.340
Pantothenic acid	-0.091	0.599	0.920	1.312	0.064	0.446	2.721	0.075	0.448	1.076
Pentadecanoic acid	-0.209	0.877	0.965	-0.086	0.991	0.994	0.015	0.985	0.997	0.011
Phenol	0.079	0.591	0.920	1.054	0.214	0.674	0.949	0.394	0.883	0.721
Phenylacetic acid	0.026	0.828	0.962	-0.331	0.898	0.974	0.026	0.975	0.997	0.112
Phenylalanine	0.655	0.028	0.350	0.626	0.109	0.496	2.760	0.072	0.448	0.868
Phenylethylamine	-1.675	0.251	0.816	-0.586	0.504	0.877	0.691	0.505	0.883	0.592
Phenyllactic acid	0.018	0.546	0.920	1.629	0.225	0.674	0.938	0.398	0.883	0.843
Phenylpyruvic acid	0.205	0.517	0.920	1.054	0.387	0.839	0.484	0.619	0.946	0.372
Phosphoenolpyruvate	-0.327	0.294	0.848	0.127	0.964	0.994	0.672	0.515	0.883	0.020
Phosphoric acid	0.865	0.435	0.914	1.811	0.000	0.001	8.891	0.000	0.028	2.382
Phosphorylethanolamine	0.377	0.331	0.869	0.901	0.392	0.839	0.503	0.607	0.946	0.387
Phytanic acid	-0.430	0.931	0.968	0.362	0.615	0.902	0.223	0.801	0.955	0.397
Phytosphingosine	-1.186	0.585	0.920	-0.690	0.620	0.902	0.199	0.820	0.960	0.355
Picolinic acid	0.139	0.651	0.936	0.656	0.881	0.971	0.101	0.905	0.977	0.092
Pimelic acid	0.350	0.967	0.986	1.367	0.188	0.644	1.156	0.322	0.818	0.863
Pinitol	-1.293	0.030	0.350	0.491	0.899	0.974	2.602	0.083	0.465	0.111
Pipecolinic acid	0.458	0.101	0.581	0.357	0.068	0.446	2.377	0.102	0.499	2.052
Proline	1.556	0.000	0.010	1.823	0.000	0.009	17.165	0.000	0.000	5.157
Propylamine	0.392	0.303	0.848	1.241	0.000	0.018	7.130	0.002	0.051	1.493

Protocatechuic acid	-0.612	0.604	0.920	0.563	0.928	0.982	0.141	0.869	0.977	0.046
Pseudouridine	0.374	0.799	0.949	0.117	0.640	0.903	0.103	0.902	0.977	0.350
Psicose	-0.371	0.528	0.920	0.429	0.653	0.903	0.572	0.568	0.928	0.291
Putrescine	0.350	0.839	0.965	-2.094	0.336	0.820	0.657	0.522	0.890	0.830
Pyridoxine	0.476	0.159	0.682	0.846	0.023	0.270	2.845	0.067	0.439	1.337
Pyrogallol	-0.694	0.046	0.381	0.321	0.326	0.819	1.452	0.243	0.729	0.572
Pyroglutamicacid	0.020	0.426	0.914	0.150	0.476	0.877	0.408	0.667	0.947	0.318
Quinic acid	-0.463	0.947	0.979	0.326	0.776	0.928	0.076	0.927	0.980	0.264
Rhamnose	1.649	0.071	0.516	0.483	0.021	0.266	2.694	0.077	0.448	2.992
Ribofuranose	0.045	0.385	0.906	0.187	0.603	0.902	0.341	0.712	0.947	0.237
Ribonic acid	-0.259	0.134	0.675	-0.532	0.511	0.877	1.194	0.311	0.795	0.470
Ribose-5-phosphate	-0.642	0.040	0.357	0.542	0.342	0.828	4.348	0.018	0.222	0.419
Ribulose	-1.448	0.012	0.192	2.650	0.259	0.708	4.904	0.011	0.171	0.934
Ricinoleic acid	-0.190	0.406	0.906	-0.126	0.561	0.902	0.990	0.378	0.883	0.411
Salicylic acid	-0.378	0.691	0.948	1.606	0.872	0.971	0.038	0.963	0.994	0.102
Sarcosine	0.837	0.327	0.869	1.695	0.098	0.493	1.473	0.238	0.729	1.499
Sedoheptulose	-0.156	0.539	0.920	0.815	0.820	0.947	0.325	0.724	0.947	0.126
Serine	0.772	0.008	0.171	0.073	0.074	0.451	5.131	0.009	0.158	0.947
Serotonin	1.182	0.016	0.225	2.514	0.022	0.266	3.511	0.037	0.338	1.407
Shikimic acid	-0.482	0.306	0.848	1.621	0.405	0.846	1.661	0.200	0.684	0.589
Sinapic acid	0.023	0.270	0.834	0.146	0.361	0.835	0.708	0.497	0.883	0.526
Sitosterol acetate	1.314	0.864	0.965	3.169	0.114	0.508	1.774	0.179	0.636	1.313
Sophorose	-1.990	0.215	0.761	-0.259	0.868	0.971	0.838	0.438	0.883	0.128
Sorbitol	-0.361	0.757	0.948	-0.371	0.594	0.902	0.402	0.671	0.947	0.400
Sorbose	-3.799	0.031	0.350	-2.203	0.611	0.902	2.765	0.072	0.448	0.522
Stearic acid	-0.481	0.086	0.544	-0.491	0.071	0.446	1.813	0.173	0.635	1.132

Stigmasterol	0.728	0.920	0.966	1.927	0.094	0.480	1.990	0.147	0.579	1.337
Succinic acid	-0.915	0.176	0.687	-0.514	0.495	0.877	0.960	0.389	0.883	0.497
Sucrose	1.420	0.983	0.993	0.195	0.975	0.994	0.001	0.999	0.999	0.017
Sulfuric acid	0.358	0.268	0.834	0.374	0.106	0.496	4.120	0.022	0.250	1.823
Sumic acid	0.488	0.397	0.906	0.309	0.840	0.957	0.392	0.677	0.947	0.157
Syringic acid	-0.385	0.475	0.914	0.431	0.203	0.674	0.884	0.419	0.883	0.945
Tagatose	-0.042	0.627	0.923	0.766	0.700	0.916	0.336	0.716	0.947	0.228
Threitol	0.053	0.601	0.920	1.958	0.002	0.063	7.231	0.002	0.051	1.971
Threonic acid	-0.070	0.080	0.523	-0.651	0.575	0.902	2.998	0.058	0.433	0.548
Threonine	0.872	0.008	0.171	0.807	0.015	0.239	5.204	0.009	0.158	1.255
Threose	0.100	0.869	0.965	0.405	0.771	0.928	0.042	0.959	0.994	0.269
Thymidine	0.162	0.230	0.773	0.698	0.117	0.515	1.520	0.228	0.729	1.116
Thymine	0.172	0.168	0.687	0.251	0.179	0.643	1.413	0.252	0.743	1.252
Trehalose	0.829	0.906	0.965	0.051	0.733	0.916	0.101	0.904	0.977	0.264
Tryptamine	-0.618	0.524	0.920	0.245	0.027	0.291	2.834	0.068	0.439	1.670
Tryptophan	-0.198	0.126	0.653	-0.301	0.314	0.796	1.481	0.237	0.729	0.596
Turanose	-0.819	0.292	0.848	0.167	0.567	0.902	0.542	0.585	0.941	0.386
Tyramine	0.323	0.573	0.920	0.445	0.224	0.674	0.822	0.445	0.883	1.031
Tyrosine	0.729	0.007	0.171	0.638	0.079	0.454	4.343	0.018	0.222	1.113
UDP N-acetylmannosamine	-1.416	0.645	0.935	-0.535	0.919	0.982	0.110	0.896	0.977	0.101
UDP-glucuronic acid	1.482	0.721	0.948	0.333	0.850	0.964	0.070	0.933	0.980	0.188
Undecanoic acid	0.768	0.915	0.965	0.428	0.255	0.703	0.624	0.540	0.910	0.792
Uracil	0.258	0.031	0.350	0.360	0.185	0.643	2.548	0.088	0.468	1.010
Urea	0.743	0.019	0.255	1.615	0.006	0.148	5.175	0.009	0.158	1.428
Uridine	-0.481	0.183	0.701	0.327	0.996	0.996	0.944	0.396	0.883	0.002
Valine	1.366	0.001	0.027	1.252	0.015	0.239	8.002	0.001	0.035	1.820

Vanillic acid	-0.195	0.773	0.948	1.123	0.142	0.574	1.604	0.211	0.706	0.981
Xylitol	2.562	0.564	0.920	1.000	0.543	0.899	0.204	0.816	0.960	0.401
Xylonic acid	0.560	0.800	0.949	0.034	0.710	0.916	0.071	0.931	0.980	0.368
Xylono-1,5-lactone	-0.414	0.152	0.682	0.157	0.208	0.674	1.127	0.331	0.834	0.711
Xylose	-0.709	0.609	0.920	-0.125	0.719	0.916	0.129	0.879	0.977	0.304

Table S8. Sensitivity analysis of key metabolites in the time-course evaluation of the COA 1201 intervention using linear mixed-effects models (LMM).

Key metabolites	Source of Variation	% of total variation	F (DFn, DFd)	*p*-value
Alanine	Time	23.3	F (2, 36) = 11.33	0.0002
Proline	Time	16.78	F (2, 36) = 5.857	0.0063

Table S9. A total of 191 metabolites identified in the group-wise comparison between the COA 1201 and placebo groups.

	COA 1201 vs Placebo (4 week)				COA 1201 vs Placebo (8 week)				ANOVA_COA 1201 vs Placebo (4 and 8 week)			
Metabolites	Log_2 (FC)	p value	FDR adjusted p value	VIP score	Log_2 (FC)	p value	FDR adjusted p value	VIP score	f value	p value	FDR adjusted p value	VIP score
2-Aminobutyric acid	0.409	0.838	0.997	0.254	1.299	0.139	0.736	1.826	0.896	0.448	0.968	1.230
2-Hydroxyhexanoic acid	0.231	0.265	0.996	1.291	2.114	0.229	0.851	1.316	0.969	0.413	0.968	1.799
3-Methylaspartic acid	-0.362	0.595	0.996	0.606	0.645	1.000	1.000	0.000	0.232	0.874	0.999	0.537
3-Phenyllactic acid	-0.352	0.849	0.997	0.267	1.228	0.858	1.000	0.273	0.037	0.990	0.999	0.473
4-Aminobutyric acid	0.639	0.333	0.996	0.931	1.300	0.013	0.723	1.919	2.515	0.066	0.714	1.600
Alanine	-0.183	0.414	0.996	0.600	0.445	0.474	0.969	0.488	0.417	0.741	0.997	0.101
Alanyl-L-alanine	-0.367	0.193	0.996	1.899	-0.208	0.321	0.914	1.064	0.982	0.407	0.968	1.740
Allopyranose	-0.675	0.314	0.996	1.781	0.702	0.256	0.857	1.682	1.124	0.346	0.968	0.754
Arabinose	-0.315	0.588	0.996	0.603	0.922	0.278	0.871	1.182	0.611	0.611	0.968	0.206
Asparagine	-3.443	0.167	0.996	1.899	0.219	0.591	0.969	0.511	0.894	0.449	0.968	0.722
Aspartic acid	-0.478	0.072	0.996	1.141	-0.112	0.695	1.000	0.273	0.946	0.424	0.968	0.831
Decanedioic acid	0.817	0.108	0.996	1.208	1.324	0.935	1.000	0.069	0.979	0.408	0.968	0.435
Decanoic acid	-0.260	0.209	0.996	1.383	1.140	0.113	0.736	1.481	1.544	0.212	0.884	1.553

Delta-Gluconolactone	-0.532	0.281	0.996	1.142	0.628	0.196	0.851	1.254	1.885	0.141	0.881	0.864
Desaminotyrosine	0.120	0.560	0.996	1.135	-0.411	0.514	0.969	1.008	0.337	0.798	0.999	0.985
Docosahexaenoic acid	0.093	0.266	0.996	0.840	1.332	0.397	0.960	0.808	0.653	0.584	0.968	0.913
Docosanoic acid	-0.326	0.445	0.996	0.422	0.731	0.609	0.969	0.355	0.766	0.517	0.968	0.326
Docosenoic acid	-0.751	0.077	0.996	1.113	0.547	0.888	1.000	0.140	0.750	0.526	0.968	0.878
Dopamine	-0.264	0.628	0.996	0.312	0.106	0.866	1.000	0.099	0.155	0.926	0.999	0.000
Elaidic acid	1.023	0.457	0.996	1.106	-0.567	0.699	1.000	0.452	0.317	0.813	0.999	0.608
Erythritol	-0.501	0.520	0.996	0.590	0.699	0.787	1.000	0.208	0.174	0.914	0.999	0.250
Erythronic acid	0.120	0.687	0.996	0.436	0.494	0.285	0.871	0.979	1.063	0.371	0.968	0.196
Erythrose	0.325	0.314	0.996	0.511	1.023	0.102	0.736	0.995	1.635	0.190	0.881	0.803
Ferulic acid	-0.200	0.191	0.996	0.861	0.680	0.372	0.957	0.549	1.087	0.361	0.968	0.379
Fructopyranose 1-phosphate	-0.191	0.539	0.996	0.421	-0.100	0.870	1.000	0.155	1.765	0.163	0.881	1.172
Fructose	0.104	0.956	0.997	0.045	0.412	0.333	0.922	0.534	0.380	0.768	0.999	0.195
Fucopyranose	0.377	0.953	0.997	0.039	0.104	0.508	0.969	0.403	0.561	0.643	0.968	0.547
Fumaric acid	-1.033	0.581	0.996	0.600	-0.019	0.524	0.969	0.565	0.282	0.839	0.999	0.586
Galactinol dihydrate	0.281	0.730	0.996	0.286	1.869	0.059	0.735	1.602	1.619	0.194	0.881	1.003
Galactitol	0.351	0.329	0.996	0.635	0.677	0.230	0.851	0.686	1.196	0.319	0.968	1.112
Galactonic acid	0.986	0.522	0.996	0.496	0.948	0.975	1.000	0.034	0.860	0.467	0.968	0.323
Galactopyranoside	-0.424	0.488	0.996	0.732	-0.366	0.202	0.851	1.225	1.557	0.209	0.884	0.581
Galactose	-2.397	0.121	0.996	2.480	-2.553	0.614	0.969	0.694	0.969	0.413	0.968	1.749
Galacturonic acid	-0.694	0.547	0.996	0.702	0.410	0.581	0.969	0.618	0.226	0.878	0.999	0.007
Gallic acid	-1.111	0.068	0.996	2.621	0.185	0.478	0.969	1.069	1.909	0.137	0.881	0.090
Gentisic acid	0.125	0.980	0.997	0.021	0.267	0.353	0.928	0.871	1.826	0.151	0.881	0.220
Gluco-2-heptulose	0.178	0.728	0.996	0.234	0.970	0.605	0.969	0.420	0.258	0.856	0.999	0.046
Glucopyranose 6-phosphate	0.991	0.969	0.997	0.045	-0.677	0.477	0.969	0.691	0.169	0.917	0.999	0.503

Glucosamic acid	-0.497	0.180	0.996	0.955	0.438	0.972	1.000	0.025	0.798	0.500	0.968	0.828
Glucose	-0.529	0.593	0.996	0.665	-0.572	0.988	1.000	0.015	0.565	0.640	0.968	0.968
Glutamic acid	-0.123	0.271	0.996	1.329	-0.099	0.293	0.871	1.023	0.810	0.493	0.968	1.549
Glutamyl-Valine	0.464	0.055	0.996	0.890	2.795	0.071	0.735	1.380	2.564	0.063	0.714	1.274
Glutaric acid	-0.573	0.372	0.996	0.969	-0.297	0.196	0.851	0.975	1.144	0.338	0.968	1.643
Glycerol	0.301	0.695	0.996	0.251	-0.711	0.032	0.723	1.410	2.223	0.094	0.782	0.534
Glycerol 3-phosphate	0.166	0.536	0.996	0.395	0.482	0.532	0.969	0.364	0.272	0.846	0.999	0.520
Glycine	0.077	0.832	0.997	0.126	-0.077	0.581	0.969	0.408	1.361	0.263	0.929	0.362
Glycolic acid	-0.049	0.737	0.996	0.152	1.565	0.501	0.969	0.460	0.809	0.494	0.968	0.066
Glycylglycine	-0.034	0.884	0.997	0.154	-0.358	0.727	1.000	0.848	5.720	0.002	0.133	3.942
Gondoic acid	-0.620	0.239	0.996	1.969	-0.958	0.043	0.723	2.490	1.771	0.162	0.881	2.928
Hexose	1.380	0.584	0.996	0.827	-1.931	0.628	0.984	0.574	0.195	0.899	0.999	0.770
Hexuronic Acid	-1.330	0.050	0.996	2.067	0.534	0.952	1.000	0.056	1.513	0.220	0.884	1.515
Histidine	3.404	0.302	0.996	1.908	0.076	0.644	0.991	0.554	0.703	0.554	0.968	1.994
Homogentisic acid	0.180	0.495	0.996	1.002	-0.766	0.548	0.969	0.775	0.288	0.834	0.999	1.038
Homoserine	0.100	0.702	0.996	0.177	0.362	0.555	0.969	0.367	0.899	0.447	0.968	0.001
Hydrocinnamic acid	-0.009	0.581	0.996	0.843	-0.988	0.752	1.000	0.455	0.136	0.938	0.999	0.744
Hydroxyglutaric acid	0.786	0.308	0.996	0.774	0.901	0.050	0.723	1.266	1.734	0.169	0.881	1.196
Hydroxyphenyllactic acid	-0.206	0.804	0.997	0.389	-0.796	0.576	0.969	0.713	0.216	0.885	0.999	0.082
Hydroxyproline	0.077	0.920	0.997	0.101	3.363	0.542	0.969	0.704	0.728	0.539	0.968	0.083
Hydroxypropionic acid	-0.177	0.677	0.996	0.387	0.118	0.949	1.000	0.052	0.088	0.966	0.999	0.158
Hypotaurine	1.095	0.380	0.996	0.868	1.656	0.026	0.723	1.724	1.892	0.140	0.881	1.805
Hypoxanthine	-0.761	0.332	0.996	1.219	2.345	0.869	1.000	0.195	0.338	0.798	0.999	0.446
Iditol	-0.277	0.584	0.996	0.472	1.995	0.019	0.723	1.936	3.056	0.035	0.603	1.015
Indoxyl sulfate	-0.566	0.327	0.996	2.295	-0.713	0.296	0.871	1.176	1.722	0.172	0.881	0.810
Inosine	-2.376	0.304	0.996	1.356	-2.272	0.746	1.000	0.386	0.542	0.656	0.971	1.435

Inositol	-0.605	0.166	0.996	0.980	-1.515	0.827	1.000	0.180	1.969	0.128	0.881	0.005
Isoleucine	-0.403	0.661	0.996	0.405	0.232	0.767	1.000	0.289	0.386	0.764	0.999	0.045
Isomaltose	1.035	0.778	0.997	0.274	1.471	0.574	0.969	0.778	0.917	0.438	0.968	0.106
Ketoisovaleric acid	0.577	0.714	0.996	0.393	2.316	0.213	0.851	1.561	0.725	0.541	0.968	1.344
Ketoleucine	0.124	0.982	0.997	0.025	-0.027	0.809	1.000	0.235	0.021	0.996	0.999	0.159
Kojic acid	-0.348	0.180	0.996	1.829	-0.153	0.595	0.969	0.683	1.028	0.386	0.968	0.970
Lactic acid	2.691	0.041	0.996	2.345	0.479	0.538	0.969	0.431	2.154	0.102	0.815	1.870
Lactitol	-0.617	0.695	0.996	0.381	-0.125	0.970	1.000	0.039	0.193	0.901	0.999	0.543
Lactobionic acid	-1.112	0.657	0.996	0.559	0.299	0.818	1.000	0.216	0.261	0.853	0.999	0.527
Lactobiose	-0.236	0.610	0.996	0.424	0.014	0.806	1.000	0.184	0.420	0.739	0.997	0.706
Lanosterin	0.212	0.981	0.997	0.033	0.868	0.295	0.871	1.400	0.526	0.666	0.972	0.572
Lauric acid	-0.422	0.641	0.996	0.535	1.601	0.049	0.723	1.706	1.458	0.235	0.884	1.108
Leucine	-0.106	0.717	0.996	0.344	-0.467	0.129	0.736	1.800	2.328	0.083	0.753	0.106
Leucrose	1.288	0.087	0.996	1.481	1.723	0.139	0.736	1.561	2.583	0.061	0.714	1.265
Levodopa	0.980	0.119	0.996	1.852	-0.547	0.752	1.000	0.360	1.037	0.382	0.968	0.932
Levoglucosan	-0.140	0.852	0.997	0.123	1.366	0.073	0.735	1.253	1.910	0.137	0.881	0.386
Linoleic acid	0.526	0.661	0.996	0.519	-0.999	0.136	0.736	1.743	0.998	0.400	0.968	0.608
Linolenic acid	0.359	0.720	0.996	0.432	-0.654	0.149	0.767	1.640	0.861	0.466	0.968	1.026
Lysine	-2.195	0.734	0.996	0.801	-1.526	0.091	0.736	2.739	0.987	0.405	0.968	1.662
Lyxose	-0.321	0.716	0.996	0.700	-0.012	0.592	0.969	0.734	1.106	0.354	0.968	2.125
Malic acid	-0.437	0.494	0.996	0.414	-0.311	0.937	1.000	0.071	0.207	0.891	0.999	0.002
Malonic acid	-0.151	0.699	0.996	0.192	1.180	0.186	0.851	1.019	0.966	0.415	0.968	0.626
Maltitol	0.123	0.465	0.996	1.250	-2.183	0.769	1.000	0.409	0.242	0.867	0.999	1.182
Maltose	0.514	0.833	0.997	0.286	-0.047	0.407	0.960	1.035	0.270	0.847	0.999	0.961
Mannose	-0.127	0.793	0.997	0.205	-0.081	0.860	1.000	0.113	0.135	0.939	0.999	0.366
Margaric acid	0.373	0.835	0.997	0.219	1.515	0.328	0.921	0.919	0.824	0.486	0.968	1.257

Melibiose	0.062	0.437	0.996	0.572	0.946	0.853	1.000	0.154	0.214	0.886	0.999	0.320
Methionine	-0.321	0.587	0.996	0.701	-0.587	0.354	0.928	1.118	2.512	0.067	0.714	0.184
Methyl 6-octadecenoate	0.061	0.736	0.996	0.117	1.225	0.425	0.965	0.501	1.654	0.186	0.881	0.038
Methylamine	-0.228	0.999	0.999	0.000	-1.224	0.107	0.736	1.167	1.682	0.180	0.881	0.548
Methylglutaric acid	0.061	0.890	0.997	0.142	0.713	0.776	1.000	0.334	0.047	0.986	0.999	0.066
Methylmalonic acid	0.214	0.201	0.996	2.500	0.474	0.755	1.000	0.570	0.758	0.522	0.968	2.498
Methylsuccinic acid	0.219	0.880	0.997	0.183	0.184	0.996	1.000	0.005	0.703	0.554	0.968	0.532
Monomyristin	-0.281	0.611	0.996	0.254	0.768	0.087	0.736	0.769	1.578	0.204	0.884	0.899
Mucic acid	0.855	0.987	0.997	0.028	-3.834	0.812	1.000	0.402	0.021	0.996	0.999	0.265
Myristic acid	0.297	0.740	0.996	0.325	0.978	0.230	0.851	1.094	0.634	0.596	0.968	1.117
N-Acetyl-Glucosamine	-0.175	0.728	0.996	0.346	-0.263	0.545	0.969	0.395	0.195	0.900	0.999	0.086
N-Acetylhexosamine	-0.115	0.938	0.997	0.055	-0.066	0.873	1.000	0.115	0.080	0.970	0.999	0.184
N-Acetyl-L-aspartic acid	-0.558	0.348	0.996	0.894	-0.086	0.376	0.957	1.025	0.570	0.637	0.968	0.215
N-Acetyl-L-leucine	0.066	0.910	0.997	0.086	1.787	0.563	0.969	0.576	1.452	0.236	0.884	0.329
N-Acetyl-mannosamine	0.065	0.947	0.997	0.052	-0.954	0.896	1.000	0.095	0.008	0.999	0.999	0.022
N-Acetylornithine	1.089	0.815	0.997	0.264	-0.826	0.404	0.960	0.740	1.005	0.397	0.968	0.322
N-Acetylputrescine	-0.035	0.881	0.997	0.068	2.388	0.022	0.723	1.726	3.359	0.024	0.603	0.824
N-Alpha-Acetyl-L-lysine	0.522	0.674	0.996	0.418	-0.350	0.399	0.960	0.790	0.648	0.587	0.968	0.362
N-Heptanoic acid	-0.168	0.710	0.996	0.238	1.736	0.049	0.723	1.484	1.800	0.156	0.881	1.105
Nicotinic acid	-0.129	0.553	0.996	0.261	-0.289	0.474	0.969	0.449	0.347	0.791	0.999	0.521
Nonadecylic acid	-0.009	0.741	0.996	0.170	1.973	0.274	0.871	0.940	0.765	0.518	0.968	0.694
Nonanoic acid	-1.785	0.257	0.996	1.733	1.329	0.218	0.851	1.430	0.970	0.412	0.968	0.198
Norepinephrine	-0.250	0.479	0.996	0.699	0.780	0.575	0.969	0.527	0.501	0.683	0.981	0.410
Norvaline	1.508	0.415	0.996	1.184	1.095	0.166	0.811	1.732	0.947	0.423	0.968	1.613
O-Acetyl-L-serine	-0.618	0.826	0.997	0.174	-1.646	0.053	0.723	1.812	2.708	0.053	0.714	1.955
Octadecanol	-0.032	0.684	0.996	0.661	0.054	0.989	1.000	0.019	0.072	0.975	0.999	0.508

Octadecatrienoic acid	1.162	0.545	0.996	0.874	-1.235	0.126	0.736	2.135	1.029	0.386	0.968	1.062
Octanedioic acid	0.093	0.740	0.996	0.159	2.759	0.099	0.736	1.337	1.870	0.144	0.881	0.694
Oleic acid	2.091	0.183	0.996	1.595	-1.731	0.641	0.991	0.461	1.206	0.315	0.968	1.212
O-Phosphorylethanolamine	-0.052	0.860	0.997	0.115	1.328	0.036	0.723	1.562	2.701	0.053	0.714	1.418
Ornithine	0.349	0.957	0.997	0.054	-1.362	0.474	0.969	0.714	0.441	0.724	0.997	0.140
Oxalic acid	0.818	0.076	0.996	3.145	0.642	0.428	0.965	1.220	2.362	0.080	0.753	1.455
Palmitic acid	0.356	0.319	0.996	0.548	0.117	0.998	1.000	0.002	1.509	0.221	0.884	0.794
Palmitoleic acid	2.614	0.305	0.996	1.058	0.694	0.350	0.928	0.741	0.724	0.541	0.968	1.285
Palmitoleyl alcohol	-0.050	0.968	0.997	0.056	0.655	0.659	1.000	0.448	0.574	0.634	0.968	0.381
Pantothenic acid	-0.567	0.145	0.996	0.835	0.650	0.891	1.000	0.107	1.419	0.246	0.885	0.946
Pentadecanoic acid	0.025	0.629	0.996	0.660	0.867	0.787	1.000	0.337	0.255	0.858	0.999	0.211
Phenol	-0.573	0.992	0.997	0.008	0.983	0.490	0.969	0.584	0.360	0.782	0.999	0.139
Phenylacetic acid	0.049	0.462	0.996	0.983	0.005	0.669	1.000	0.455	0.261	0.853	0.999	0.846
Phenylalanine	-0.273	0.413	0.996	0.551	-0.259	0.534	0.969	0.480	0.490	0.690	0.984	0.437
Phenylethylamine	-1.575	0.148	0.996	1.486	0.475	0.911	1.000	0.111	0.674	0.571	0.968	0.870
Phenylpyruvic acid	0.253	0.320	0.996	0.448	0.936	0.413	0.963	0.572	0.627	0.601	0.968	0.501
Phosphoric acid	0.067	0.864	0.997	0.223	0.499	0.217	0.851	0.894	3.963	0.012	0.488	0.735
Phytosphingosine	-2.186	0.148	0.996	1.944	-3.047	0.042	0.723	2.792	2.453	0.071	0.718	3.352
Pimelic acid	0.290	0.818	0.997	0.252	1.776	0.113	0.736	1.391	1.189	0.321	0.968	0.625
Pinitol	-0.836	0.711	0.996	0.492	1.007	0.273	0.871	1.451	1.235	0.304	0.968	0.172
Pipecolinic acid	0.168	0.472	0.996	0.925	-0.583	0.244	0.851	1.415	0.846	0.474	0.968	1.894
Proline	-0.099	0.612	0.996	0.689	0.456	0.220	0.851	1.478	0.609	0.611	0.968	1.395
Propylamine	0.392	0.107	0.996	1.706	0.581	0.255	0.857	0.483	6.204	0.001	0.133	0.127
Protocatechuic acid	-0.213	0.648	0.996	0.203	0.796	0.918	1.000	0.078	0.155	0.926	0.999	0.338
Pseudouridine	0.154	0.453	0.996	0.950	0.302	0.429	0.965	0.782	0.436	0.728	0.997	0.937
Psicose	0.369	0.610	0.996	0.414	1.999	0.009	0.723	2.374	3.130	0.032	0.603	1.835

Putrescine	4.622	0.087	0.996	2.602	-0.667	0.599	0.969	0.625	1.227	0.307	0.968	1.225
Pyridoxine	-0.377	0.717	0.996	0.376	-0.152	0.736	1.000	0.283	0.481	0.697	0.986	0.854
Pyrogallol	-0.453	0.625	0.996	0.449	0.663	0.959	1.000	0.044	0.506	0.680	0.981	0.198
Pyroglutamic acid	0.100	0.891	0.997	0.065	0.016	0.749	1.000	0.179	0.081	0.970	0.999	0.151
Rhamnose	1.761	0.576	0.996	1.451	-0.691	0.861	1.000	0.361	0.317	0.813	0.999	0.132
Ribofuranose	-0.208	0.221	0.996	0.520	0.149	0.899	1.000	0.080	0.594	0.621	0.968	0.027
Ribofuranose 5-phosphate	-0.709	0.021	0.996	1.367	0.773	0.245	0.851	0.679	3.603	0.018	0.577	0.873
Ribonic acid	1.538	0.841	0.997	0.224	0.736	0.904	1.000	0.118	0.417	0.742	0.997	0.549
Ribulose	-6.254	0.023	0.996	4.038	3.212	0.071	0.735	2.304	3.228	0.028	0.603	1.110
Ricinoleic acid	0.221	0.629	0.996	0.506	-0.362	0.069	0.735	1.894	1.429	0.243	0.885	0.781
Salicylic acid	0.054	0.494	0.996	0.259	1.930	0.305	0.884	1.044	0.804	0.496	0.968	1.051
Sarcosine	-3.293	0.860	0.997	0.312	1.890	0.159	0.800	1.776	0.552	0.649	0.968	0.972
Serine	-0.506	0.643	0.996	0.408	-0.486	0.495	0.969	0.440	1.057	0.374	0.968	0.016
Serotonin	-0.413	0.713	0.996	0.331	1.975	0.387	0.960	0.744	0.331	0.803	0.999	0.370
Shikimic acid	0.300	0.813	0.997	0.216	1.799	0.216	0.851	1.315	1.463	0.233	0.884	0.094
Sinapic acid	1.056	0.445	0.996	0.808	-0.732	0.782	1.000	0.268	0.577	0.632	0.968	0.149
Sitosterol acetate	-0.169	0.953	0.997	0.075	2.524	0.124	0.736	2.057	1.227	0.307	0.968	0.913
Sophorose	-0.216	0.729	0.996	0.260	-1.200	0.691	1.000	0.496	0.356	0.785	0.999	0.223
Sorbitol	-0.114	0.861	0.997	0.164	-0.386	0.292	0.871	1.101	0.533	0.661	0.972	0.461
Sorbose	-0.315	0.927	0.997	0.110	0.395	0.510	0.969	0.935	1.487	0.227	0.884	0.441
Stearic acid	0.089	0.473	0.996	0.982	0.343	0.817	1.000	0.244	0.223	0.880	0.999	0.339
Stigmastanol acetate	0.008	0.884	0.997	0.101	2.291	0.805	1.000	0.288	0.973	0.411	0.968	0.849
Stigmasterol	0.138	0.428	0.996	0.921	0.803	0.591	0.969	0.577	1.887	0.141	0.881	1.983
Succinic acid	-0.588	0.141	0.996	1.434	0.037	0.700	1.000	0.346	0.792	0.503	0.968	1.206
Sucrose	0.840	0.577	0.996	0.654	-0.369	0.969	1.000	0.034	0.152	0.928	0.999	0.284
Syringic acid	0.001	0.776	0.997	0.312	0.820	0.689	1.000	0.468	0.618	0.606	0.968	0.748

Threitol	-0.637	0.923	0.997	0.094	1.454	0.099	0.736	1.316	3.900	0.013	0.488	0.208
Threonic Acid	0.362	0.520	0.996	0.686	-0.624	0.480	0.969	0.986	2.503	0.067	0.714	1.145
Threonine	-0.393	0.405	0.996	0.613	-0.335	0.244	0.851	0.698	0.710	0.550	0.968	0.759
Thymidine	-0.580	0.113	0.996	1.396	0.034	0.995	1.000	0.007	0.645	0.589	0.968	0.774
Thymine	0.018	0.683	0.996	0.482	0.329	0.910	1.000	0.120	0.078	0.972	0.999	0.256
Trehalose	0.412	0.925	0.997	0.137	-0.694	0.478	0.969	0.896	0.182	0.908	0.999	0.577
Tryptamine	-0.174	0.344	0.996	1.037	-0.309	0.704	1.000	0.363	1.670	0.182	0.881	1.737
Tryptophan	-0.816	0.111	0.996	1.095	-1.472	0.001	0.244	2.296	5.475	0.002	0.133	2.313
Turanose	0.505	0.404	0.996	0.694	0.892	0.986	1.000	0.015	0.606	0.614	0.968	0.004
Tyramine	-0.174	0.285	0.996	1.313	0.638	0.933	1.000	0.090	0.428	0.733	0.997	0.875
Tyrosine	-0.330	0.835	0.997	0.168	-0.442	0.123	0.736	1.176	0.957	0.419	0.968	0.748
UDP N-acetylmannosamine	-0.433	0.816	0.997	0.273	-0.326	0.789	1.000	0.375	0.052	0.984	0.999	0.002
UDP-glucuronic acid	2.520	0.976	0.997	0.049	-0.777	0.865	1.000	0.260	0.053	0.984	0.999	0.448
Undecanoic acid	1.264	0.946	0.997	0.083	1.345	0.108	0.736	1.165	0.682	0.566	0.968	0.462
Uracil	-0.251	0.408	0.996	0.490	-0.127	0.396	0.960	0.735	0.720	0.544	0.968	0.514
Urea	0.658	0.035	0.996	1.548	1.215	0.101	0.736	1.225	3.149	0.031	0.603	1.298
Uridine	-0.237	0.645	0.996	0.318	0.275	0.706	1.000	0.289	0.427	0.734	0.997	0.304
Valine	-0.226	0.688	0.996	0.290	0.098	0.894	1.000	0.120	1.076	0.366	0.968	0.536
Vanillic acid	-0.084	0.898	0.997	0.105	0.922	0.355	0.928	0.931	0.907	0.443	0.968	0.186
Xylitol	-0.500	0.347	0.996	1.637	-1.152	0.459	0.969	0.903	0.553	0.648	0.968	1.366
Xylonic acid	5.052	0.039	0.996	2.935	1.479	0.784	1.000	0.364	2.291	0.087	0.753	0.573
Xylono-1,5-lactone	0.196	0.689	0.996	0.348	1.669	0.235	0.851	0.907	0.657	0.582	0.968	1.023
Xylose	-0.428	0.558	0.996	0.710	0.379	0.913	1.000	0.141	0.123	0.946	0.999	0.184

Table S10. Sensitivity analysis of key metabolites in the group-wise comparison between the COA 1201 and placebo groups using linear mixed-effects models (LMM).

Target metabolites	Source of Variation (Effect)	F value (DFn, DFd)	*p*-value
Tryptophan	Interaction (Time x Group)	F (2, 95) = 0.3829	0.683
	Time (week)	F (1, 95) = 7.222	0.009
	Group (COA vs Placebo)	F (2, 95) = 0.7627	0.469
Lactic acid	Interaction (Time x Group)	F (2, 95) = 4.144	0.019
	Time (week)	F (1, 95) = 0.03604	0.850
	Group (COA vs Placebo)	F (2, 95) = 0.01711	0.983
Phytospingosine	Interaction (Time x Group)	F (2, 95) = 1.581	0.211
	Time (week)	F (1, 95) = 0.4063	0.525
	Group (COA vs Placebo)	F (2, 95) = 2.082	0.130

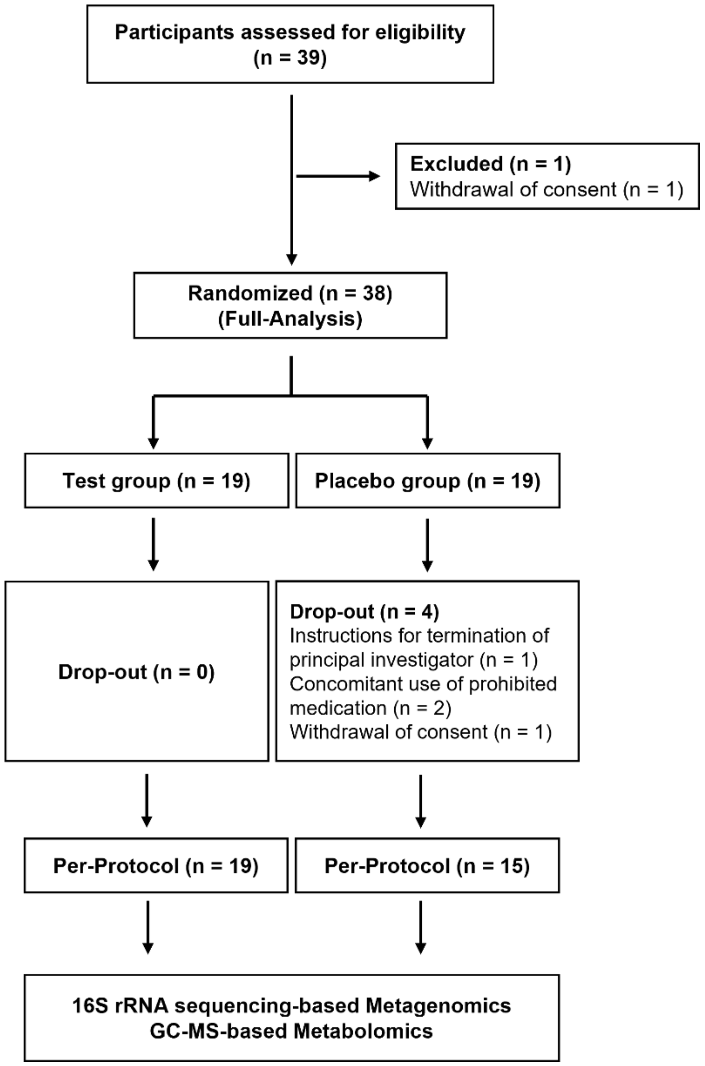

Figure S1. CONSORT diagram.

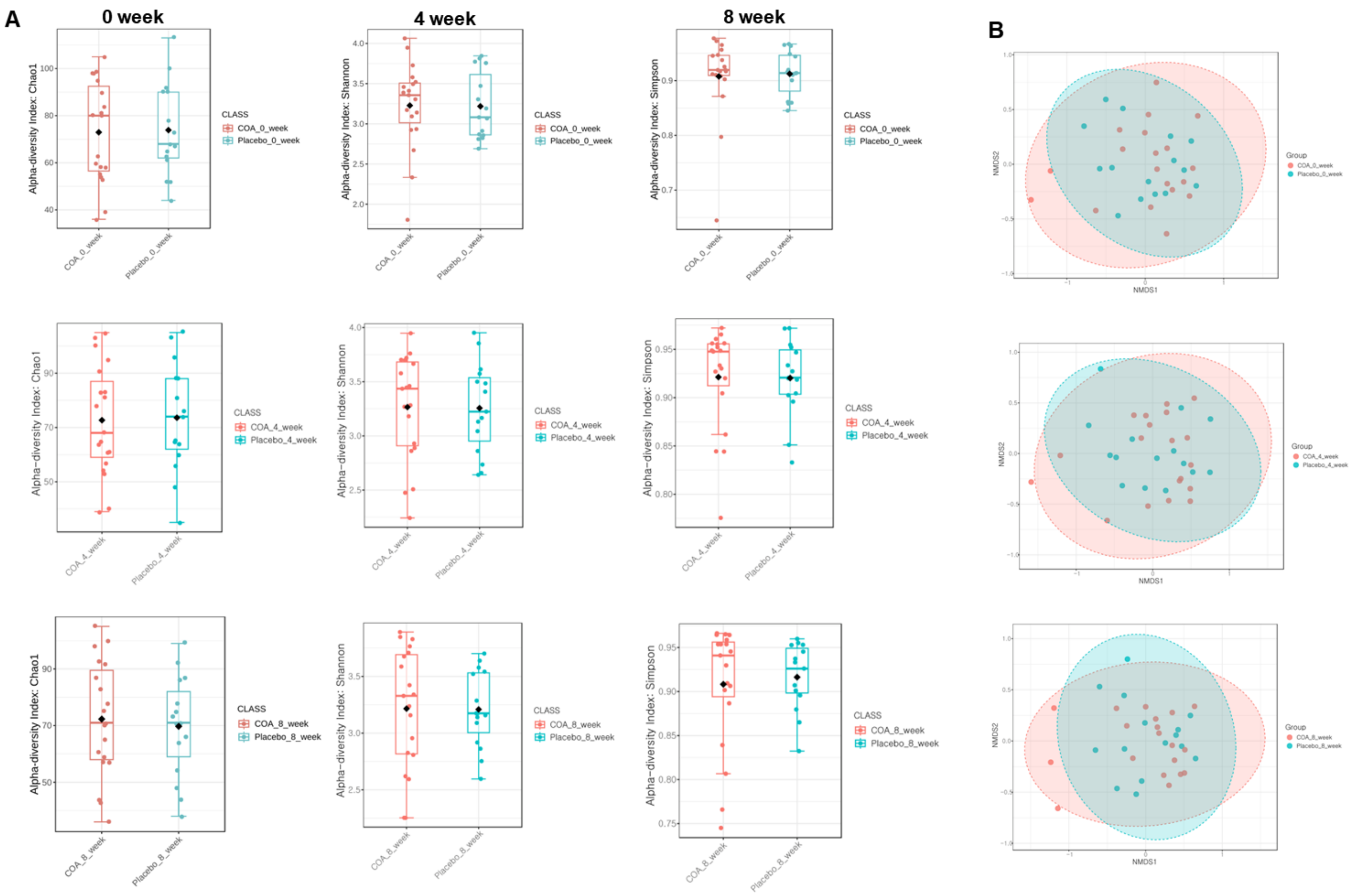

Figure S2. (A). Alpha-diversity indices (Chao1, Shannon, Simpson) of fecal microbiota in placebo and COA 1201 groups at 0, 4, and 8 weeks, analyzed using Mann–Whitney U-test. (B) Beta-diversity based on non-metric multidimensional scaling (NMDS) and Bray–Curtis dissimilarity index of fecal microbiota in placebo and COA 1201 groups at 0, 4, and 8 weeks.

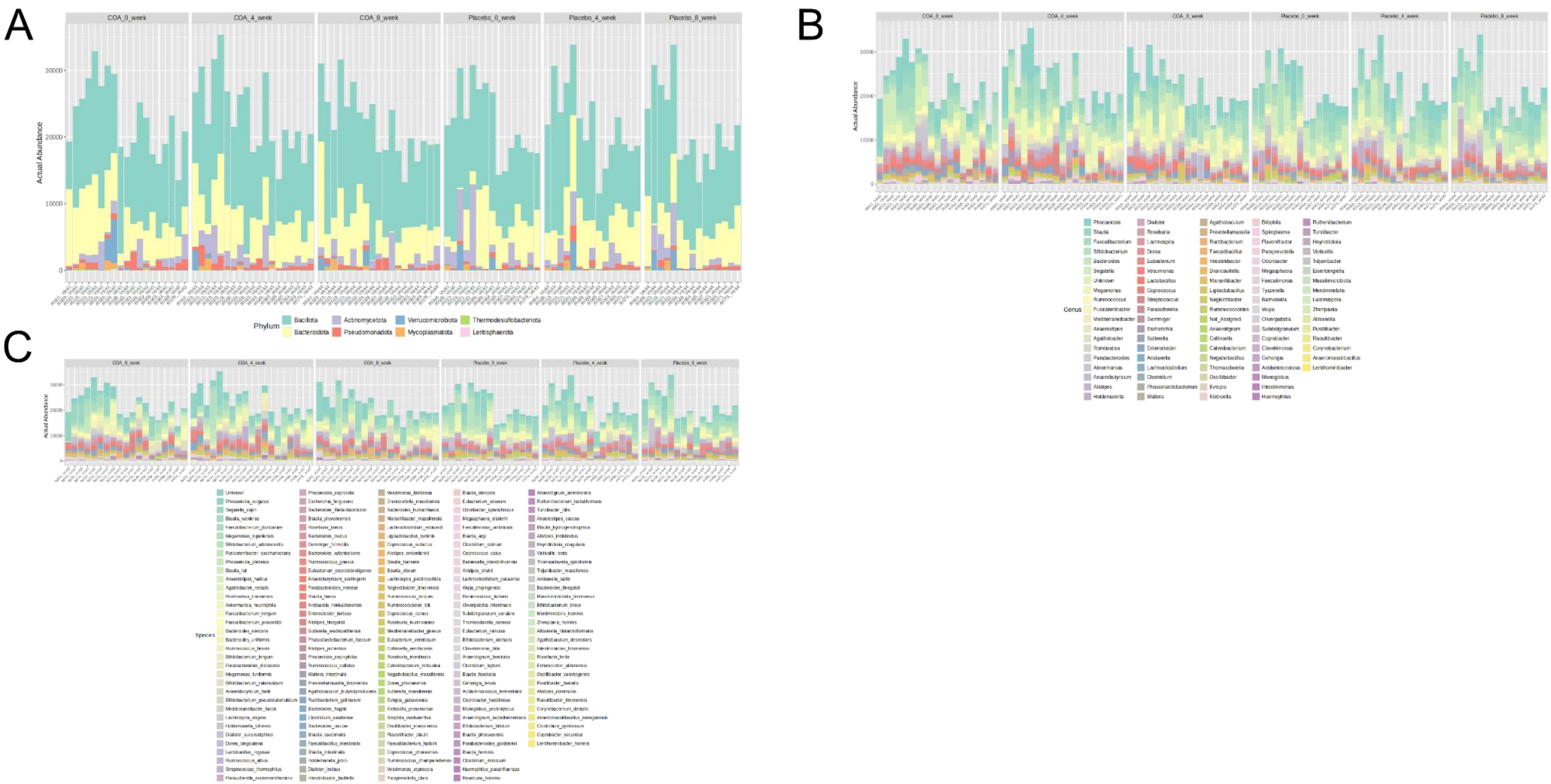

Figure S3. (A) Phylum, (B) genus, and (C) species-level enrichment analysis between placebo and COA groups.
